# Addressing the implementation challenge of risk prediction model due to missing risk factors: The submodel approximation approach

**DOI:** 10.1002/sim.10184

**Published:** 2024-09-12

**Authors:** Tianyi Sun, Allison B. McCoy, Alan B. Storrow, Dandan Liu

**Affiliations:** 1 Department of Biostatistics, Vanderbilt University Medical Center, Nashville, Tennessee; 2 Department of Biomedical Informatics, Vanderbilt University Medical Center, Nashville, Tennessee; 3 Department of Emergency Medicine, Vanderbilt University Medical Center, Nashville, Tennessee

**Keywords:** clinical prediction model, electronic health records, implementation, missing risk factors

## Abstract

Clinical prediction models have been widely acknowledged as informative tools providing evidence-based support for clinical decision making. However, prediction models are often underused in clinical practice due to many reasons including missing information upon real-time risk calculation in electronic health records (EHR) system. Existing literature to address this challenge focuses on statistical comparison of various approaches while overlooking the feasibility of their implementation in EHR. In this article, we propose a novel and feasible submodel approach to address this challenge for prediction models developed using the model approximation (also termed “preconditioning”) method. The proposed submodel coefficients are equivalent to the corresponding original prediction model coefficients plus a correction factor. Comprehensive simulations were conducted to assess the performance of the proposed method and compared with the existing “one-step-sweep” approach as well as the imputation approach. In general, the simulation results show the preconditioning-based submodel approach is robust to various heterogeneity scenarios and is comparable to the imputation-based approach, while the “one-step-sweep” approach is less robust under certain heterogeneity scenarios. The proposed method was applied to facilitate real-time implementation of a prediction model to identify emergency department patients with acute heart failure who can be safely discharged home.

## 1 ∣ INTRODUCTION

Clinical prediction models can provide evidence-based guidance and are useful for healthcare provider’s decision making, if implemented appropriately. Research has been conducted to investigate and enhance methods for prediction model development and validation, which has built a solid foundation for its utility. However, for real-time implementation in clinical practice, there are still challenges to be addressed to maximize its functionality. Less than half of evidence based models have been implemented due to various challenges such as other competing demands from frontline providers, lack of knowledge, skills and resources, and misalignment of research evidence with operational priorities.^1^ The rate of implementing clinical prediction models in electronic health records (EHR) system is even lower, although a large number of such models are published in the medical literature each year.^2^ One major roadblock in EHR implementation of clinical prediction model is the requirement of prompt real-time data extraction and risk calculation with minimal computation resources in the presence of missing risk factors.^3^

For example, STRATIFY^4^ is a risk prediction model with 13 risk factors developed from a multi-center prospective cohort study to identify low-risk patients with acute heart failure (AHF) who can be safely discharged home from the emergency department (ED). The goal of this model is to aid emergency care physicians to make disposition decisions for patients with AHF such that low risk patients can be safely discharged home. For real-time implementation, the availability of all 13 risk factors at the time of disposition decision making is essential. The STRATIFY implementation study aims to implement this risk prediction model at multiple EDs. Preliminary EHR data extraction of STRATIFY risk factors revealed more than 30% of AHF patients had at least one missing STRATIFY risk factor at the time of disposition decision making, and thus cannot have STRATIFY risk calculated in its original form. To maximize the utility of clinical prediction models, statistical methods that can address such missing risk factors challenge in individual patient’s risk prediction and are feasible to implement are warranted.

There are three general statistical approaches to handle missing risk factors for individual patient risk prediction: (1) submodel approaches,^5^ (2) imputation approaches,^6-9^ and (3) the marginalization approach.^7^ The submodel approaches aim to develop models based on non-missing risk factors only. For such an approach, the most straightforward solution is to fit all the submodels according to specific missing patterns which requires access to the original data used to develop the prediction model. An alternative strategy was proposed by Marshall et al^5^ to estimate submodel coefficients based on the asymptotic distribution of the original model coefficient estimates which is generally not publicly available. Imputation approaches aim to impute missing risk factors for individual patients. Both naive imputation methods such as zero imputation, mean imputation, subgroup mean imputation and advanced imputation methods such as stacked multiple imputation have been discussed in Janssen et al^6^ in the context of predicting risk for a new patient. Hoogland et al^7^ further proposed to use the fixed chained equations for imputation, which is a single imputation approach and can be considered as a simplification of traditional imputation by chained equations with the stochastic component removed. It was showed that the imputation by fixed chained equations performed better than other imputation-based approaches including the stacked multiple imputation approach. Similar conclusion was drawn in Sisk et al^10^ when comparing regression-based single imputation and multiple imputation. The marginalization approach^7^ aims to predict the marginal risk given the non-missing risk factors by integrating out the missing risk factors using Monte Carlo integration approximation and was shown to have intermediate model performance comparing to existing submodel approaches and imputation approaches.^7^

Although there are substantial statistical literature on each of the above approaches, their feasibility in real world setting vary. The submodel approaches are generally feasible to implement in EHR system as they only require basic logical operation to select submodels based on observed missing patterns of individual patients and subsequent arithmetic operation of risk prediction. On the contrary, the imputation approaches are not readily available to be integrated in electronic health system, despite the existence of many software to apply advanced imputation approaches in statistical analysis. This is confirmed by Health IT teams across multiple EDs in the STRATIFY implementation study. Due to constraints on computation resources and technical capacity, even implementing some sophisticated single imputation approaches such as fixed chained equations is very challenging for health care systems. As a consequence, only naive imputation approaches such as mean-imputation have been adopted in clinical practice to facilitate prediction model implementation.^11^ Similar to the imputation approaches, the marginalization approaches are also not feasible to be integrated in EHR due to the required computation resources. Therefore, we strategically focus on developing better submodel approaches to address missing risk factor challenges.

In this study, we propose a novel submodel approach where the submodel coefficients can be estimated by correcting the coefficients from the original prediction model. The proposed submodel estimation is based on prediction models developed using a model approximation approach. While traditional risk prediction model selection is against the observed outcome, both Harrell et al^12^ and Paul et al^13^ proposed an idea of model selection based on an approximated outcome. This model approximation approach includes two steps: (1) obtain an approximated outcome that is a continuous latent measurement of the observed outcome and, (2) perform the model selection against the approximated outcome. In Paul et al,^13^ such approximated outcome is termed as the “preconditioning” outcome. The proposed submodel approach first estimates the preconditioning outcome using the original model coefficients and then projects the estimated preconditioning outcome onto the range space of non-missing risk factors. The advantage of the proposed submodel approach is that it only requires the original model coefficients and a dataset with all risk factors from a cohort representing the target population where the prediction model will be implemented. Comprehensive simulations were conducted to evaluate our proposed approach and we included the existing “one-step-sweep” (OSS) submodel approach^5^ as well as the imputation by fixed chained equations approach^7^ as comparators. Since our proposed approach can borrow information from the target population, we expect it has better adaptability in the presence of heterogeneous underlying populations between the original cohort used to develop the prediction model and the target population aimed to implement the prediction model. The article focuses on developing submodels for binary outcomes. However, the same approach can be applied to any type of outcomes where a preconditioning outcome can be obtained.

This article is organized as follows. In Section 2, we describe the details of the proposed submodel approach. In Section 3, we conduct comprehensive simulations. In Section 4, we apply this approach to develop submodels of STRATIFY risk prediction model^4^ which will be implemented as an EHR-based clinical decision support tool to aid disposition decision making for acute heart failure patients. In Section 5, we provide a summary and discuss the advantages and limitations of our proposed approach.

## 2 ∣ METHODS

### 2.1 ∣ Preconditioning outcome

The idea of using preconditioning outcome for model selections and prediction model development has been discussed and applied in multiple studies including the motivating STRATIFY study.^4,12,13^

Let Y denote the binary outcome and Z denote the vector of all candidate risk factors. Using the preconditioning method, the risk prediction model is developed with two steps. The first step is to obtain a consistent estimator of the outcome, namely the preconditioning outcome $\tilde{Y}$ that truly represents the underlying latent value of Y given Z. Several studies discussed approaches to obtain $\tilde{Y}$, which is usually the continuous linear predictor from a saturated model. Harrell et al^12^ provided a thorough discussion about using penalized maximum likelihood estimation to estimate the $\tilde{Y}$. Bair et al^14^ proposed to use supervised principal components to predict $\tilde{Y}$. Paul et al^13^ further discussed the advantage of using supervised principal components to obtain the preconditioning outcome. The second step is to select final risk factors X from Z against the continuous preconditioning outcome $\tilde{Y}$ using standard model selection approaches such as stepwise selection or LASSO.

Using the stepwise regression, coefficient estimates under the preconditioning approach has a simple format. For a dataset with n subjects, let X be a *p*-vector variable and X be the corresponding design matrix. In addition, let $\tilde{Y}$ denotes the *n*-vector of preconditioning outcome. The coefficient estimates under the preconditioning approach is thus the least squares estimator

(1) $${\hat{\beta }}^{\text{pre}}={\left(X^{T}X\right)}^{-1}X^{T}\tilde{Y}.$$

The predicted risk for subject i with risk factor $x_{i}$ is thus ${\mathrm{logit}}^{-1}(x_{i}^{T}{\hat{\beta }}^{\text{pre}})$.

One advantage of performing model selection with preconditioning outcome is that such procedure can mitigate the effect of noisy predictors under the presence of a large pool of candidate risk factors. By separating the steps of model approximation and selection using the preconditioning outcome, the model selection result can yield a consistent set of risk factors.^13^

### 2.2 ∣ Preconditioning-based submodels

Let W denote a subset of X with q risk factors. For simplicity of the notation, we let both X and W include an unit for intercept. For a patient with complete information only for W, the preconditioning-based submodel satisfies

(2) $$E[\tilde{Y}|W]=W^{T}\theta^{\text{pre}},$$

where $\theta^{\text{pre}}$ is a vector with the first element being the intercept.

Intuitively, one could obtain an estimator for $\theta^{\text{pre}}$ by directly fitting W against the preconditioning outcome $\tilde{Y}$. However, this approach works only when the preconditioning outcome $\tilde{Y}$ and the subset of risk factors W from the original data used to develop the prediction model are available, which is rare in practice. In most cases, only ${\hat{\beta }}^{\text{pre}}$, the estimated coefficients from the original prediction model, are made publicly available.

In the following, we show that an unbiased estimator for $\theta^{\text{pre}}$ can be derived without $\tilde{Y}$ and only using W, X and ${\hat{\beta }}^{\text{pre}}$. Let W denote the design matrix for W with the first column being unit vector for intercept. Since W is a subset of X, it follows from simple linear algebra operation that W=HW, where $H=X{\left(X^{T}X\right)}^{-1}X^{T}$ is the hat matrix of X and $H^{T}=H$. Therefore, the least squares estimator for $\theta^{\text{pre}}$ in Equation (2) is

(3) $${\hat{\theta }}^{\text{pre}}={\left(W^{T}W\right)}^{-1}W^{T}\tilde{Y}\;\;={\left(W^{T}W\right)}^{-1}W^{T}H\tilde{Y}\;\;={\left(W^{T}W\right)}^{-1}W^{T}X{\left(X^{T}X\right)}^{-1}X^{T}\tilde{Y}\;\;={\left(W^{T}W\right)}^{-1}W^{T}X{\hat{\beta }}^{\text{pre}}.$$

The proposed estimator only requires W and X from a representative cohort in addition to ${\hat{\beta }}^{\text{pre}}$. When implementing an existing clinical prediction model in an EHR system, it is often necessary to validate the model using EHR data pulled from the target population. Therefore, W and X are often available.

Furthermore, let $\bar{W}=X\setminus W$ and $\bar{W}$ denote the corresponding n×(p−q) matrix. We can partition ${\hat{\beta }}^{\text{pre}}$ as $\left({\hat{\beta }}_{W}^{\text{pre}},{\hat{\beta }}_{\bar{W}}^{\text{pre}}\right)$. It follows that $\tilde{Y}={\tilde{Y}}_{W}+{\tilde{Y}}_{\bar{W}}=W{\hat{\beta }}_{W}^{\text{pre}}+\bar{W}{\hat{\beta }}_{\bar{W}}^{\text{pre}}$, where ${\tilde{Y}}_{W}$ and ${\tilde{Y}}_{\bar{W}}$ are two components of the preconditioning outcome corresponding to non-missing risk factor W and missing risk factor $\bar{W}$ of a particular missing pattern. Then, $\theta^{\text{pre}}$ can be rewritten as

(4) $${\hat{\theta }}^{\text{pre}}={\left(W^{T}W\right)}^{-1}W^{T}(W,\bar{W})({\hat{\beta }}_{W}^{\text{pre}},{\hat{\beta }}_{\bar{W}}^{\text{pre}})\;\;={\hat{\beta }}_{W}^{\text{pre}}+{\left(W^{T}W\right)}^{-1}W^{T}\bar{W}{\hat{\beta }}_{\bar{W}}^{\text{pre}}\;\;={\hat{\beta }}_{W}^{\text{pre}}+{\left(W^{T}W\right)}^{-1}W^{T}{\tilde{Y}}_{\bar{W}}.$$

Therefore, ${\hat{\theta }}^{\text{pre}}$ is equivalent to the corresponding original prediction model coefficients plus a correction factor which is another ordinary least square estimator of non-missing risk factors regressed against the missing risk factor component of the preconditioning outcome. In other words, the correction factor is the solution that forms the orthogonal projection of the preconditioning outcome component of missing risk factor ${\tilde{Y}}_{\bar{W}}$ on to the range space of non-missing risk factors W. Please note that this derivation does not require any missingness assumptions.

One advantage of the proposed method is that it can borrow information from the target population by using X and W from a representative cohort of the target population. In general, it’s expected that some heterogeneity may exist in the underling patient population between the original cohort and the target population. Even if the model validation of the original prediction model for the target population might show satisfactory discrimination and calibration, such heterogeneity can still potentially affect the submodel performance. Therefore, we expect the performance of our proposed submodel approach to be more robust in the presence of heterogeneous underlying populations compared to other approaches due to its information borrow feature.

### 2.3 ∣ OSS-based submodels

An alternative approach of fitting submodels with a binary outcome is the one-step sweep (OSS) method,^5^ where the prediction model is developed by fitting a logistic regression,

(5) $$\mathrm{logit}\;\text{Pr}(Y=1|X)=X^{T}\beta^{\text{oss}},$$

with selected risk factors X against the binary outcome Y. The MLE estimator ${\hat{\beta }}^{\text{oss}}$ follows an asymptotic multivariate normal distribution ${\hat{\beta }}^{\text{oss}}∼N(\beta^{\text{oss}},V)$, where V is the variance-covariance matrix. We can partition ${\hat{\beta }}^{\text{oss}}$ as $\left({\hat{\beta }}_{W}^{\text{oss}},{\hat{\beta }}_{\bar{W}}^{\text{oss}}\right)$. Then the coefficients in the submodel

$$\mathrm{logit}\;\text{Pr}(Y=1|W)=W^{T}\theta^{\text{oss}},$$

can be estimated based on the conditional distribution of ${\hat{\beta }}_{W}^{\text{oss}}|{\hat{\beta }}_{\bar{W}}^{\text{oss}}=0$. Specifically,

$${\hat{\beta }}_{W}^{\text{oss}}|{\hat{\beta }}_{\bar{W}}^{\text{oss}}=0∼N(\beta_{W}^{\text{oss}}-V_{W\bar{W}}V_{\bar{W}\;\bar{W}}^{-1}\beta_{\bar{W}}^{\text{oss}},V_{WW}-V_{W\bar{W}}V_{\bar{W}\;\bar{W}}^{-1}V_{\bar{W}W}),$$

where $V_{\mathrm{WW}}$, $V_{W\bar{W}}$, $V_{\bar{W}W}$ and $V_{\bar{W}\bar{W}}$ are the partitioning of V corresponding to $\beta_{W}^{\text{oss}}$ and $\beta_{\bar{W}}^{\text{oss}}$. Based on such conditional distribution, the submodel estimator can be written as:

(6) $${\hat{\theta }}^{\text{oss}}={\hat{\beta }}_{W}^{\text{oss}}-{\hat{V}}_{W\bar{W}}{\hat{V}}_{\bar{W}\;\bar{W}}^{-1}{\hat{\beta }}_{\bar{W}}^{\text{oss}},$$

where ${\hat{V}}_{W\bar{W}}$ and ${\hat{V}}_{\bar{W}\bar{W}}$ are partitions of the estimated covariance matrix $\hat{V}$.

The advantage of OSS approach is that the submodel can be approximated without additional data collection. However, the covariance matrix of ${\hat{\beta }}^{\text{oss}}$ from the original data is required and usually it is not publicly available. In addition, this approximation only depends on the information from the original cohort and thus is not expected to be as robust to the heterogeneous target population as the proposed approach.

## 3 ∣ SIMULATION STUDIES

### 3.1 ∣ Simulation setup

In the simulation studies, we assessed the performance of the proposed submodel approach and compared it with the OSS-based submodel as well as the fixed chained equations imputation approach.^7^ Comprehensive simulations were conducted in a target cohort with or without heterogeneity from the original cohort used to develop the prediction model. Therefore, two datasets were simulated with the first dataset mimicking the original data for prediction model development and the second dataset representing an external cohort from the target population for prediction model implementation. For all approaches, the first dataset was used to obtain the original prediction model and relevant information such as the estimated covariance matrix of the coefficients estimates for use by the OSS-based approach. To develop and test the proposed submodel approach, we split the second dataset into the training dataset and the testing dataset. For preconditioning-based submodel approach, the design matrices of W and X from the training dataset were implemented to obtain the estimated coefficients and later the submodel performance was assessed using the testing dataset. For OSS-based approach, the estimated coefficients can be directly calculated using the estimated coefficients and covariance from the original prediction model and similarly the submodel performance was evaluated in the testing dataset. For imputation-based approach, the imputation models were first developed using training dataset and then the original model performance was assessed in the testing dataset.

The first dataset included 20 000 observations. To mimic the STRATIFY model, we simulated 13 continuous variables from a multivariate normal distribution with covariance matrix Σ, where Σ was obtained using the original STRATIFY data. Then, X was generated by having the last four elements dichotomized to binary risk factors. To measure the correlation involving binary risk factors, polyserial and polychoric correlations were calculated from STRATIFY data. The risk factors distribution and true values of coefficients are presented in Table S1 (from the supporting information), where larger absolute values of true coefficients represent greater importance in risk score calculation. The true prediction model under the preconditioning approach is based on a linear model for the continuous preconditioning outcome, whereas the true prediction model under the OSS method is based on a logistic regression model. In order to simulate the original dataset (ie, the first dataset) such that it satisfies the linear format of the true prediction model under both methods, we related the preconditioning outcome and the corresponding binary outcome through a bridge distribution.^15^ Specifically, the preconditioning outcome $\tilde{Y}=X^{T}\beta^{\text{pre}}+\omega$, where ω∼Bridge(ϕ=0.980). The parameter of the bridge distribution ϕ was chosen to allow 90% of the variance of $\tilde{Y}$ be explained by X which is a commonly used cutoff for $R^{2}$ in model selection under the preconditioning approach. The binary outcome was derived as Y=1 if $\tilde{Y}+\epsilon -c>0$ and Y=0 otherwise, where ϵ∼logistic(0,1). Under this setting, logit $P(Y=1|X)=\phi *(X^{T}\beta^{\text{pre}})=X^{T}\beta^{\text{oss}}$. Therefore, the simulated original dataset satisfies the true models under both methods. The cutoffs c=5.25,3.64 correspond to low (10%) and moderate (30%) event rates. After the first dataset was simulated, the ${\hat{\beta }}^{\text{pre}}$ and ${\hat{\beta }}^{\text{oss}}$ were separately estimated. Theoretically, the preconditioning outcome is a continuous latent variable that will be approximated and utilized for model selection. Since the focus of this study is to evaluate the proposed submodel approach, our simulations avoided the steps of model selection, and instead directly obtained the true prediction models using the simulated preconditioning outcome. In this case, the original prediction models under both preconditioning- and OSS-based approaches are comparable.

The second dataset was simulated with 5000 observations with some heterogeneities in the distribution of X, and later split into a training dataset with 4000 observations and a testing dataset with 1000 observations. We considered three types of heterogeneity: heterogeneous mean for continuous risk factors ($X_{3}\text{-}X_{6}$), heterogeneous variance for continuous risk factors ($X_{3}\text{-}X_{6}$), and heterogeneous prevalence for binary risk factors ($X_{10}\text{-}X_{13}$). The scenario without any heterogeneity was also included and considered as the reference (see details in Table S2. Using the training dataset, the original prediction models are first validated and recalibrated as needed. Then, the preconditioning- and OSS-based submodels were derived respectively using the validated model. The imputation models including all risk factors from the original model and the observed outcome were fitted. Using the testing dataset, the preconditioning- and OSS-based submodel performance were assessed. For imputation-based approach, the original prediction model performance was evaluated by incorporating imputed values.

In this simulation study, we first explored missing one risk factor scenarios. Additionally, we considered three scenarios missing two risk factors, including missing two continuous risk factors ($X_{3}$, $X_{4}$), one continuous plus one binary risk factors ($X_{3}$, $X_{10}$), and two binary risk factors ($X_{12}$, $X_{13}$). The performance was evaluated using measures of discrimination (C-index), calibration (calibration-in-the-large and calibration slope) and accuracy.^16^ The accuracy was measured by negative predicted value (NPV), Brier score and root mean squared prediction error (rMSPE). NPV is the probability of not having an event among low risk patients and it was calculated among observations with predicted risk lower than 3%. It is the most critical performance measure for STRATIFY since the ultimate goal of STRATIFY is to identify low risk AHF patients for safe ED discharge. All measures were calculated across all scenarios, except for NPV which was only assessed under the scenario where the event rate was low (10%). For each scenario, a total of 1000 simulations were conducted. The distribution of these performance measures was visualized using violin plots to provide direct comparison across multiple scenarios. We repeated the entire simulation under the scenarios where the sample sizes of the training dataset and the testing dataset are 800 and 200. We also repeated the entire simulation but with the noise term ω following normal distribution to assess the robustness of the proposed approach to the choice of noise distribution.

### 3.2 ∣ Simulation results

C-indices do not seem to differ between the preconditioning-based submodels, the OSS-based submodels and the imputation by fixed chained equations approaches across any scenarios (Figure S1). Under all three approaches, C-indices vary with heterogeneous variance scenarios but do not vary with the other two types of heterogeneity scenarios.

**FIGURE 1:**
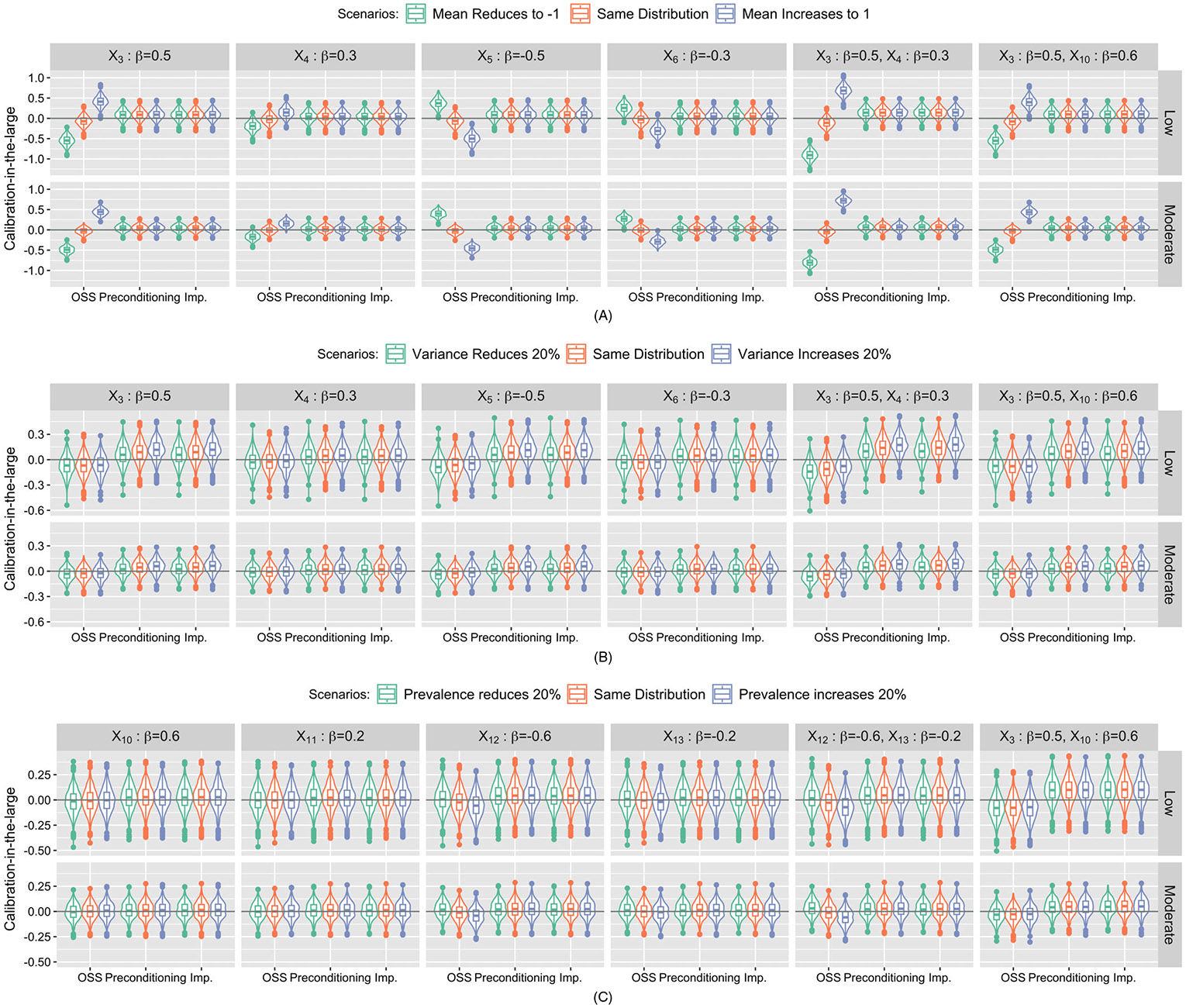
Simulation study results: Violin plots of calibration-in-the-large under three heterogeneity scenarios: (A) Heterogeneous mean, (B) heterogeneous variance and (C) heterogeneous prevalence from submodel- and imputation-based approaches corresponding to missing one or two continuous/binary risk factors. Each column represents missing risk factors with varying importance (standardized coefficient) in the risk calculation. *X*_3_-*X*_6_ represent continuous risk factors, while *X*_10_-*X*_13_ represent binary risk factors, respectively. The first row represents scenarios with low event rate (10%). The second row represents scenarios with moderate event rate (30%). The gray horizontal line at zero represents the ideal calibration-in-the-large.

Results of calibration-in-the-large were summarized in Figure 1. Under the scenarios that missing one risk factor, the distributions of calibration-in-the-large from preconditioning-based submodels and the imputation approach are fairly stable and do not vary with any heterogeneity scenarios and importance of missing risk factors or event rates. For OSS-based submodels, with low event rate, higher mean of the missing continuous risk factor in the testing dataset results in larger calibration-in-the-large (Figure 1A). This pattern is opposite for the protective factors ($X_{5}$ and $X_{6}$). In addition, the pattern is stronger for more important missing risk factors ($X_{3}$ and $X_{5}$). Similar patterns are observed for moderate event rate scenarios. Under heterogeneous variance, the distributions of calibration-in-the-large from OSS-based submodels are fairly stable but tends to be smaller comparing to the other two approaches. Under heterogeneous prevalence scenarios, the distributions of calibration-in-the-large from OSS-based submodels slightly decrease with increasing prevalence when missing an important binary risk factor ($X_{12}$). For other missing one binary risk factor scenarios, the distributions are fairly stable and comparable with the other two approaches. When missing two risk factors, the distributions of calibration-in-the-large from preconditioning-based submodel and imputation approach remain fairly stable. For OSS-based submodel, the variation in the distributions of calibration-in-the-large across heterogeneous mean scenarios is more distinct for the submodel missing two continuous risk factors ($X_{3}$, $X_{4}$).

In general, the distributions of calibration slope from all three approaches are comparable (Figure S2). Under heterogeneous variance scenarios, calibration slopes from all three approaches tend to slightly decrease with increased variance. Such variation tends to be more obvious in calibration slopes from OSS-based approach. Under heterogeneous mean or prevalence scenarios, the distributions of calibration slopes are fairly stable.

**FIGURE 2:**
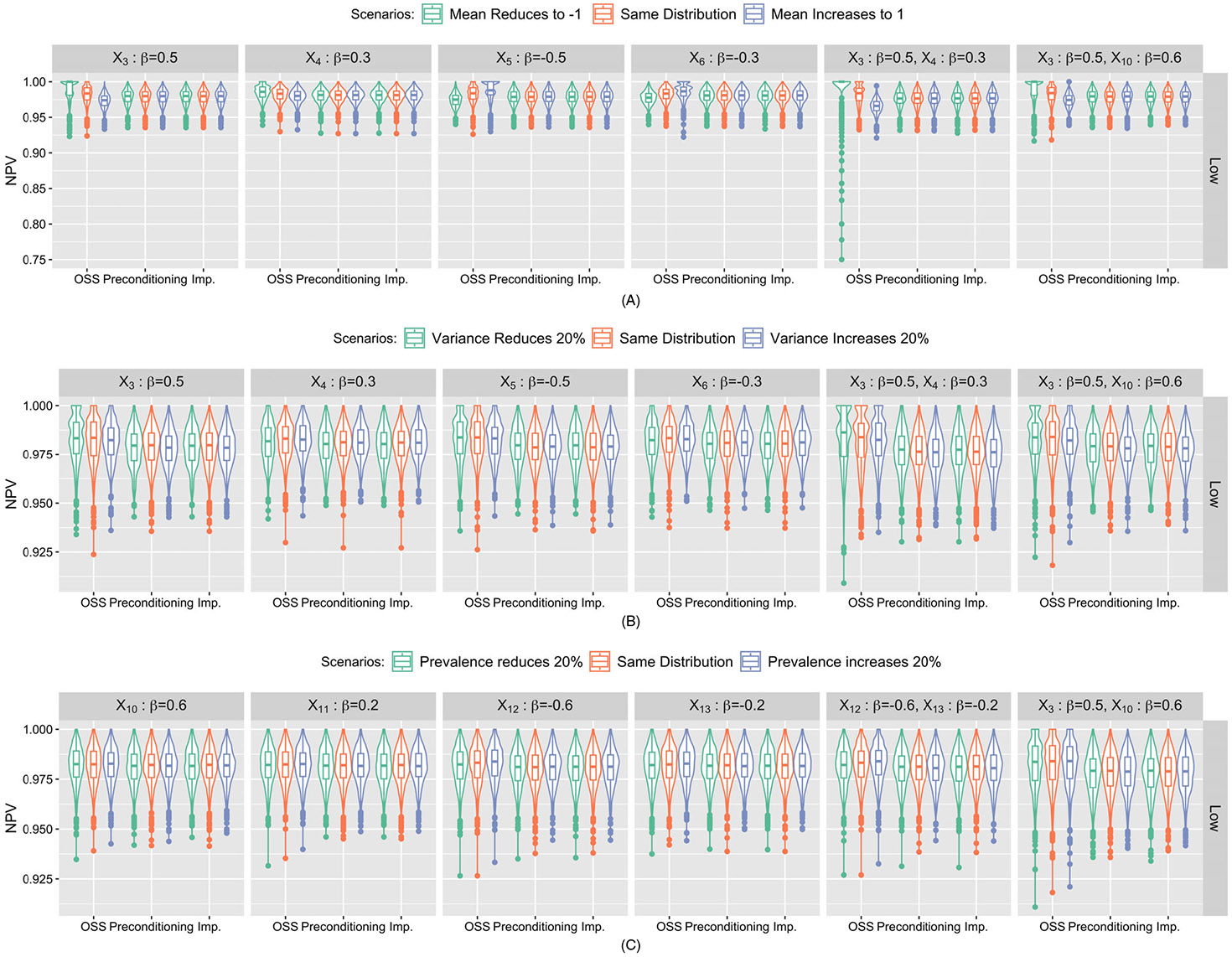
Simulation study results: Violin plots of NPV under three heterogeneity scenarios: (A) Heterogeneous mean, (B) heterogeneous variance and (C) heterogeneous prevalence from submodel- and imputation-based approaches corresponding to missing one or two continuous/binary risk factors. Each column represents missing risk factors with varying importance (standardized coefficient) in the risk calculation. *X*_3_-*X*_6_ represent continuous risk factors, while *X*_10_-*X*_13_ represent binary risk factors, respectively. The row represents scenarios with low event rate (10%).

NPV results were summarized in Figure 2. When missing one risk factor, for preconditioning-based submodels and the imputation approach, the distributions of NPV are fairly stable and do not vary with any heterogeneity scenarios and importance of missing risk factors or event rates. For OSS-based submodels, NPVs decrease with increased mean of the missing risk factor ($X_{3}$ and $X_{4}$, Figure 2A). As expected, this pattern is reversed for protective factor ($X_{5}$ and $X_{6}$). For the other two types of heterogeneity scenarios, the distributions of NPV are fairly stable and comparable with the other two approaches. When missing two risk factors, the distributions of NPV from preconditioning-based submodel and imputation-based approach remain stable. For OSS-based submodel, the variation in the distributions of NPV across heterogeneous mean scenarios is more distinct, especially for the submodel missing two continuous risk factors.

Brier score results were summarized in Figure S3. When missing one risk factor, under the heterogeneous mean scenarios, Brier scores from the preconditioning-based submodels and the imputation approach are quite stable. For OSS-based submodels, Brier scores increase with the heterogeneity in mean. This pattern is most obvious under the moderate event scenarios for more important missing risk factors ($X_{3}$ and $X_{5}$). Under heterogeneous variance or heterogeneous prevalence scenarios, all three approaches are comparable where the distributions of Brier score vary across heterogeneity scenarios in the same direction. When missing two risk factors, similar patterns in the distributions of Brier score are observed for preconditioning-based submodel and imputation-based approach. For OSS-based submodel, the variation in distributions across heterogeneous mean scenarios increases for the submodel missing two continuous risk factors.

**FIGURE 3:**
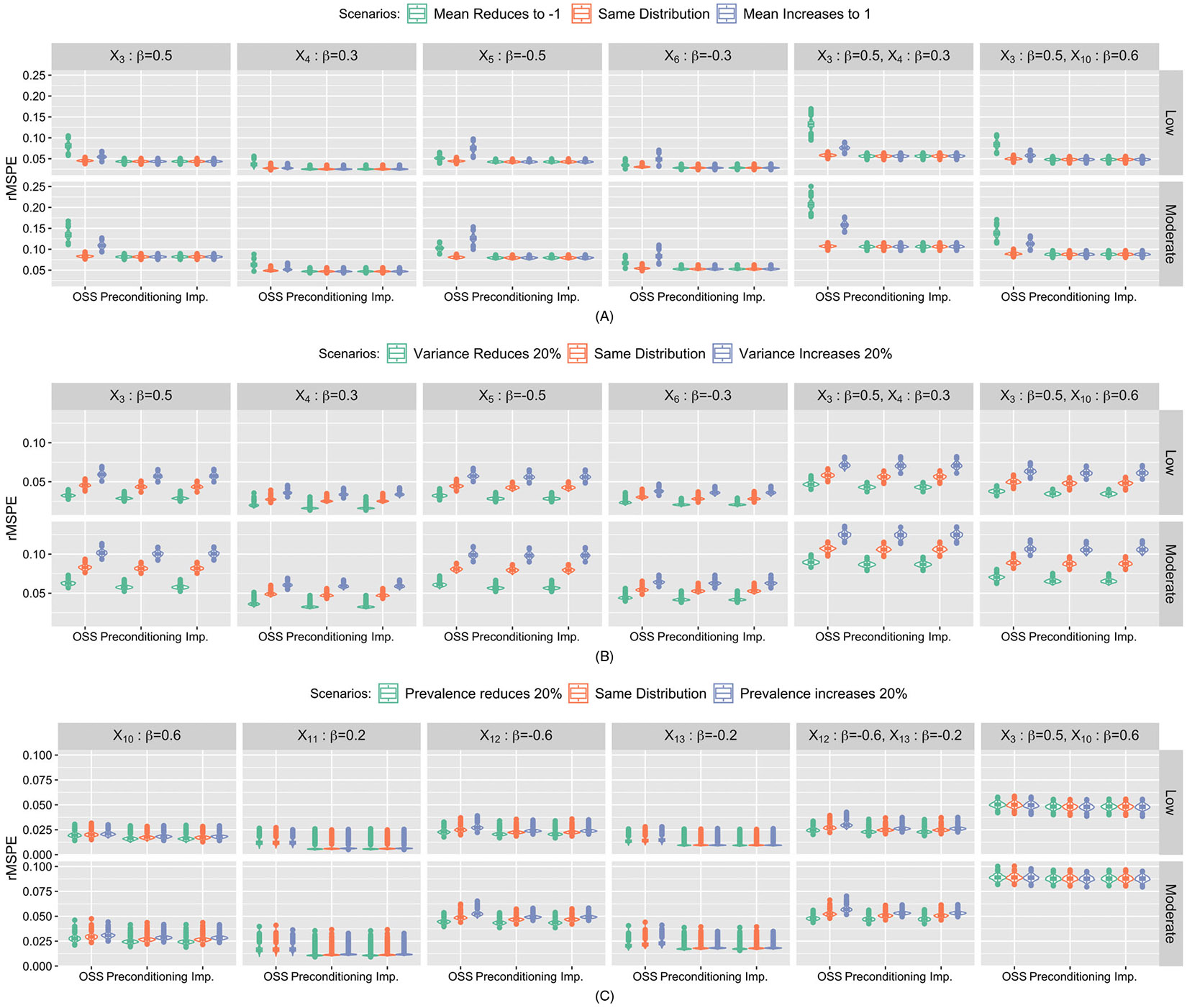
Simulation study results: Violin plots of rMSPE under three heterogeneity scenarios: (A) Heterogeneous mean, (B) heterogeneous variance and (C) heterogeneous prevalence from submodel- and imputation-based approaches corresponding to missing one or two continuous/binary risk factors. Each column represents missing risk factors with varying importance (standardized coefficient) in the risk calculation. *X*_3_-*X*_6_ represent continuous risk factors, while *X*_10_-*X*_13_ represent binary risk factors, respectively. The first row represents scenarios with low event rate (10%). The second row represents scenarios with moderate event rate (30%).

Results of rMSPE were summarized in Figure 3. When missing one risk factor, the rMSPEs from OSS-based submodels are generally larger than the other two approaches. Under the heterogeneous mean scenarios (Figure 3A), rMSPEs from preconditioning-based submodels and the imputation approach are quite stable, whereas rMSPEs from OSS-based submodels increase with the heterogeneity in mean. Under the heterogeneous variance scenarios, those three approaches are comparable where the distributions of rMSPE vary across heterogeneous variances. Under the heterogeneous prevalence scenarios, rMSPEs from all three approaches slightly vary across multiple scenarios with the pattern being more obvious for the important risk factors ($X_{10}$ and $X_{12}$). On the other hand, the distributions of rMSPE from preconditioning-based submodels and the imputation approach tend to have smaller variance across heterogeneous prevalence scenarios. When missing two risk factors, similar patterns are detected for preconditioning-based submodel and the imputation-based approach. For OSS-based submodel, the variation in distributions of rMSPE across heterogeneous mean scenarios increases for the submodel missing two continuous risk factors.

Similar results were observed for all measures under smaller sample size of the second dataset as well as the condition that the noise term ω follows normal distribution.

## 4 ∣ APPLICATION

### 4.1 ∣ STRATIFY prediction model

The STRATIFY prediction model was developed to identify low risk patients with acute heart failure who can be safely discharged home. The primary outcome was 30-day AHF related adverse events including (ordered by severity) death, cardiopulmonary resuscitation, mechanical cardiac support, mechanical ventilation, emergent dialysis, acute coronary syndrome and emergency revascularization. Due to the presence of high dimensional data challenge, the preconditioning method was used for model selection. To derive the preconditioning outcome, the first eight principal components from the 57 candidate risk factors were obtained and used to characterize the risk of mortality and serious complications. The linear combination of those eight principal components, namely the preconditioning outcome here, was then used for backward model selection. Using the selection criteria that 90% variance of preconditioning outcome can be explained by selected risk factors, 13 risk factors which are commonly available within 3 h of ED presentation are finally selected into the original STRATIFY model. The selected risk factors includes age, four vital signs (body mass index, diastolic blood pressure, respiratory rate, arterial oxygen saturation), four ED lab tests (B-type natriuretic peptide, blood urea nitrogen, sodium, troponin-I), three therapy/medication histories (on dialysis therapy, on outpatient supplemental oxygen, on outpatient angiotensin-converting enzyme inhibitor) and prolonged QRS duration.

### 4.2 ∣ STRATIFY implementation in EHR

The proposed submodel approach was adopted to facilitate the implementation of STRATIFY prediction model^4^ as an EHR-based clinical decision support tool at Vanderbilt University Medical Center. EHR data including all STRATIFY risk factors, the timestamps when these risk factors were entered in EHR, and 30-day STRATIFY outcomes were extracted for AHF patients who were admitted to the Vanderbilt ED between January 1, 2019 and December 31, 2021. Patients without chronic heart failure were excluded since patients with new onset of heart failure disease are usually not considered for home discharge and thus are not target population of STRATIFY. Patients with emergency severity index^17^ of one were also excluded as those patients are too ill to be considered for home discharge. Ideally, the extracted EHR data have complete information on all STRATIFY risk factors, so the proposed submodel approach can be directly applied. However, missing data is inevitable in EHR data. To handle missingness in the data where the proposed submodel approach was going to be applied, we integrated the multiple imputation procedure to develop and assess the proposed submodel approach.

The workflow of developing and assessing the proposed submodel involved three majors steps and was illustrated in Figure S4. Here, the EHR data was split into a training dataset (n=3176) and a testing dataset (n=794) stratified by the adverse event. Within the training dataset, 10 imputed datasets were generated. In the first step, the pooled estimates of calibration-in-the-large as well as calibration slope were obtained and tested for significance with standard errors calculated using Rubin’s rules. The full model can be unchanged or recalibrated using the pooled estimates based on the validation result. In the second step, the same 10-imputed training datasets were used. The coefficients from preconditioning-based submodels were obtained within each imputed dataset using the validated full model coefficients from the first step and then pooled across imputed datasets. In the third step, 10 imputed testing datasets were generated. The model performance measures from the validated full model (from the first step) as well as the developed submodels (from the second step) were calculated in each imputed dataset and pooled across 10 imputations. Using the similar strategy, we additionally assessed the performances of OSS-based submodel as well as the imputation-based approach. For imputation-based approach, the full model was evaluated by incorporating imputed values.

All possible missing risk factors combinations with at most three missing risk factors were considered. As age and the three therapy/medication histories won’t be missing during the EHR data extraction, only patterns corresponding to missing other nine risk factors were considered. The model performance were assessed using C-index, calibration-in-the-large and calibration slope, NPV, and the Brier score. The use of NPV is critical since the STRATIFY model aims to identify low-risk AHF patients for safe discharge. Here, NPV is calculated using the 5th percentile of predicted risk as the cutoff. The 95% confidence intervals were obtained for all performance measures. For C-index, NPV and Brier score, the 95% confidence interval was constructed using 100 bootstrap replicates. To identify eligible submodels that can be potentially adopted for EHR integration, we calculated the averaged NPV of 1st to 10th percentiles of predicted risk and considered the averaged NPV from the validated full model as the gold standard. The submodels with averaged NPV greater or equal to the one from the full model were first selected. In the clinical practice, we allow the risk score to be updated when some missing risk factors become available. Therefore, we restricted the selection of submodels to those submodels whose nested missing patterns also satisfied the averaged NPV criteria. This is to avoid the case where a risk score can be calculated with a certain missing pattern but cannot be calculate when some missing risk factors become available.

### 4.3 ∣ Results

**FIGURE 4:**
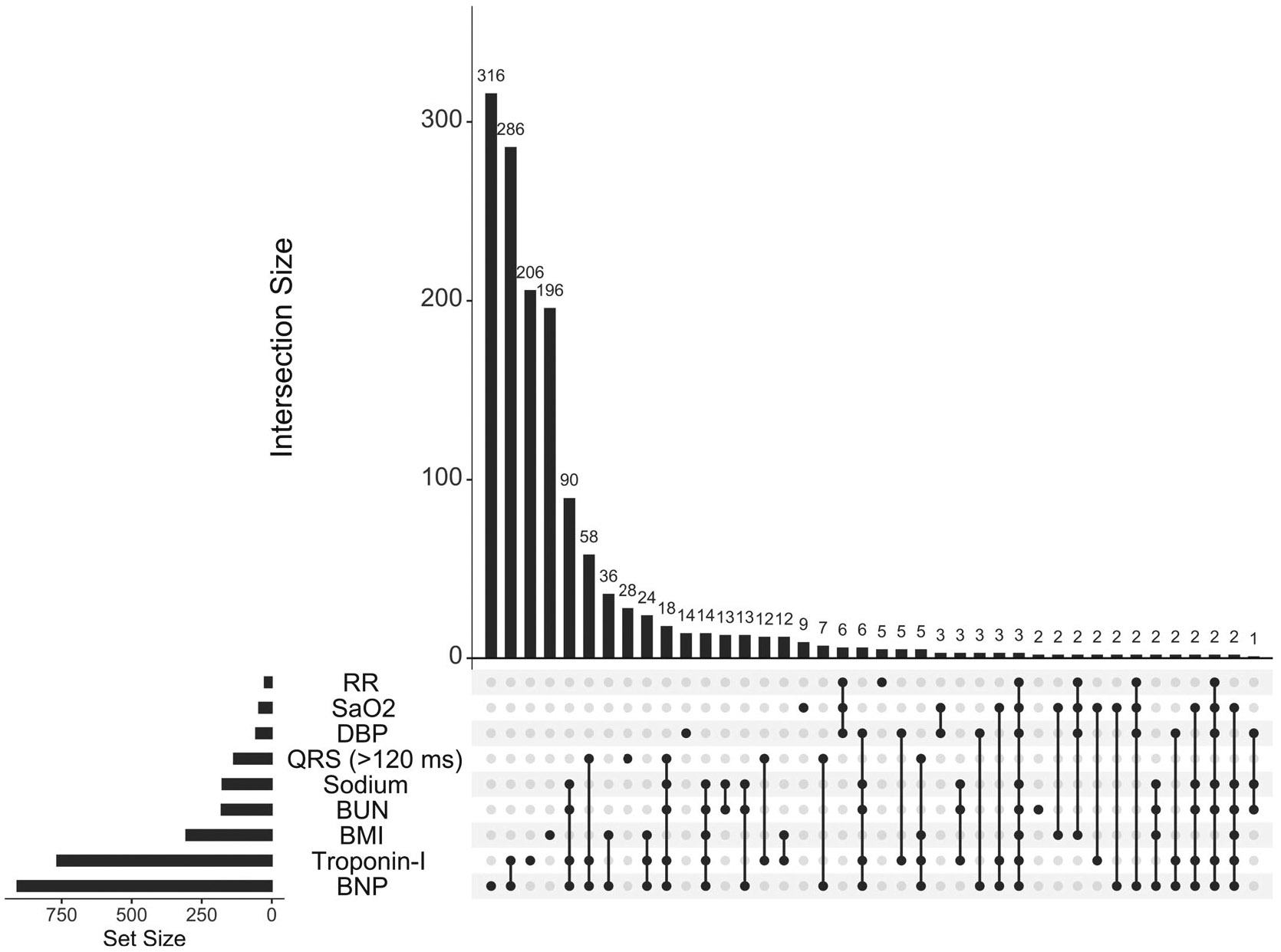
Missing patterns of STRATIFY risk factors among acute heart failure patients admitted to the emergency department at VUMC.

Baseline patient characteristics including STRATIFY risk factors are summarized in Table 1. A total of 3970 patients were included in the analysis with median age of 67 years old, 53.3% male, 25.5% African American and median BMI of 29.3. A total of 1030 (25.9%) patients have at least one 30-day STRATIFY adverse event. Patients with 30-day STRATIFY adverse events are more likely to be male and tend to have lower BMI and diastolic blood pressure, as well as higher levels of BNP, BUN and troponin-I. In addition, patients experiencing any adverse event are less likely to receive outpatient medications such as ACE inhibitor, and more likely to have QRS duration greater than 120. Other characteristics are similar comparing two groups. There are 1434 (36.1%) patients having at least one missing STRATIFY risk factor. The missing frequencies of individual risk factors range from 0.6% to 22.9% with respiratory rate having the lowest missingness and BNP having the highest missingness (see missing patterns in Figure 4).

Based on the validation using the training dataset, both intercept and slope from the original STRATIFY model were recalibrated. This is expected because the event rate from the retrospectively collected EHR data is much higher than the 12% event rate from the original STRATIFY cohort which requires patients’ consent. After that, submodels corresponding to 129 missing patterns were developed. Assessed using the testing dataset, the C-index, NPV and Brier score from the validated full model are 0.62, 0.90 and 0.19, respectively. The calibration in-the-large is −0.04 (95%CI: −0.21-0.12) and calibration slope is 0.93 (95% CI: 0.56-1.30).

The performance of preconditioning-based submodels are summarized in Table2. Among 129 submodels, 80 are selected with eligible performance, where C-index ranges from 0.60 to 0.63, NPV ranges from 0.82 to 0.92 and Brier score ranges from 0.19 to 0.19. The confidence intervals of calibration in-the-large and slope from all selected submodels include zero and one respectively. Due to the long list of submodels, Table 2 only presents the performance of full model (ie, the gold standard) and a subset of submodels corresponding to observed missing patterns from the extracted EHR data. The eligible submodels are summarized at the top of Table 2. By adopting the proposed submodel approach, 864 (60.3%) out of 1434 patients missing some risk factors can have their predicted risk calculated at the time of ED admission. Results based on all 129 missing scenarios are included in the supporting information (Table S3). Besides, under most missing scenarios, the NPV performance from preconditioning-based submodel are generally comparable to those from OSS-based submodel and the imputation-based approach. The performances comparing all three approaches are available upon request.

## 5 ∣ DISCUSSION

Motivated by the implementation of a risk prediction model for a target population where risk prediction for a new patient is often subject to missing risk factors, we proposed a novel submodel approach for prediction models developed with model approximation approach.^12,13^ The proposed submodel coefficients estimator was shown to be equivalent to the corresponding coefficients estimator from the original model plus a correction factor. Comparing to the existing OSS submodel approach,^5^ the performance of the proposed submodel was shown to be more robust to various types of heterogeneity in risk factor distributions between the target cohort and the original cohort. Such advantage is expected, as the proposed estimator can further correct the estimated coefficients through borrowing information from the target population. This correction is in addition to the potential recalibration that may occur during the model validation process.

In the simulation study, the model performances from the proposed submodel approach and the imputation by fixed chained equations approach are very similar across all scenarios. To further explore the relationship between these two approaches, we plugged the proposed estimator (4) into the Equation (2) as

$$\hat{E}(\tilde{Y}|W)=W^{T}{\hat{\beta }}_{W}^{\text{pre}}+W^{T}{\left(W^{T}W\right)}^{-1}W^{T}\bar{W}{\hat{\beta }}_{\bar{W}}^{\text{pre}}.$$

Here, ${\left(W^{T}W\right)}^{-1}W^{T}\bar{W}$ is equivalent to the least square estimator from the linear imputation model for missing risk factors $\bar{W}$ using observed risk factors W, which is specified as $E(\bar{W}|W)=W^{T}\gamma$. The corresponding imputed values of $\bar{W}$ is $W^{T}{\left(W^{T}W\right)}^{-1}W^{T}\bar{W}$. Therefore, the proposed submodel approach can be considered as a special case of imputation approach. Thus, the predict risks from two approaches are almost equivalent.

When integrating a statistical approach into an EHR system, feasibility of implementation is the most crucial factor. The proposed submodel takes this into consideration and only requires estimated coefficients from the original prediction model and a dataset of all selected risk factors from the target population. Without additional computational burden, the risk score can be prompt calculated by simple arithmetic operation once the corresponding submodel is identified. For OSS-based submodel, although the coefficients can be approximated without inquiring either the original dataset or an external dataset, it does require the estimated covariance matrix from the original model which can be rarely found from the literature. For the imputation by fixed chained equations approach, the imputation models can be developed in advance which saves massive computational cost. However, since the outcome is consistently absent for a new patient, it becomes necessary to impute at least two variables using chained equations. Consequently, the iterative procedure, which tends to be more complicated for EHR integration, is always required. In summary, based on the feasibility of model development and EHR implementation, the proposed preconditioning-based submodel approach is a more realistic option for real-time risk prediction given that a new patient has some risk factors missing.

One challenge in applying statistical methods including the proposed submodel approach in EHR data is to handle missingness. Please note that this missing data challenge is about missing data in analytical dataset used to develop submodels and is different from the missing data challenge for individual patient risk prediction that the proposed submodel will address. Such challenge is typically coped with multiple imputation.^7^ In the application section using Vanderbilt EHR data, we demonstrated the procedures of developing and assessing the proposed submodel with multiple imputed datasets (see details in Figure S4). This procedure was adopted for the real STRATIFY implementation.

The proposed submodel approach has a limitation. As stated in the method section, the proposed approach relies on the use of preconditioning outcome which is not the most commonly used model selection strategy for prediction model development. Nonetheless, the advantage of the proposed approach borrowing information from the target population can be generalized to other model selection strategies. For example, for binary outcome, Neuhaus et al^18^ showed the coefficients from the marginal model can be approximated as a function of coefficients from the conditional model and the first two moments of the covariates to be marginalized over. In this case, the conditional model, marginal model and marginalized covariates are analogous to the original model, submodel and missing risk factors considered in this article. Therefore, the information of missing risk factors from the target population can be borrowed to approximate submodel coefficients. This work is currently under development.

In conclusion, many risk prediction models have been developed, but only a few have been implemented in clinical practice due to various challenges including the presence of missing risk factors.^3^ Statistical methods such as the proposed approach can address some of these issues and are warranted to support the implementation of many existing prediction models.

## Supplementary Material

Appendix S2: Supplemental Material.

Appendix S1: Supplemental Material.

Additional supporting information can be found online in the Supporting Information section at the end of this article.

## Figures and Tables

**TABLE 1:** The baseline characteristics among acute heart failure patients admitted to the emergency department at VUMC.

	Any 30-day adverse event		
	No (N = 2940)	Yes (N = 1030)	Total (N = 3970)
**Age, years**			
Median [IQR]	67.0 [56.0, 77.0]	68.0 [57.0, 77.0]	67.0 [57.0, 77.0]
**Sex**			
Female	1415 (48.1%)	440 (42.7%)	1855 (46.7%)
Male	1525 (51.9%)	590 (57.3%)	2115 (53.3%)
**Race**			
White	2090 (71.1%)	718 (69.7%)	2808 (70.7%)
Black	745 (25.3%)	268 (26.0%)	1013 (25.5%)
Other	24 (0.8%)	7 (0.7%)	31 (0.8%)
Missing	81 (2.8%)	37 (3.6%)	118 (3.0%)
**BMI**			
Median [IQR]	29.7 [24.8, 36.8]	28.6 [24.1, 33.9]	29.3 [24.6, 36.2]
Missing	211 (7.2%)	94 (9.1%)	305 (7.7%)
**BNP (pg/ml)**			
Median [IQR]	586 [194, 1280]	938 [361, 2250]	662 [235, 1500]
Missing	655 (22.3%)	253 (24.6%)	908 (22.9%)
**DBP (mm Hg)**			
Median [IQR]	77.0 [65.0, 91.0]	75.0 [63.0, 90.0]	77.0 [64.0, 91.0]
Missing	41 (1.4%)	15 (1.5%)	56 (1.4%)
**Sodium (mmol/L)**			
Median [IQR]	139 [136, 141]	138 [135, 141]	139 [136, 141]
Missing	122 (4.1%)	54 (5.2%)	176 (4.4%)
**Respiratory rate**			
Median [IQR]	18.0 [17.0, 22.0]	19.0 [17.0, 22.0]	18.0 [17.0, 22.0]
Missing	18 (0.6%)	7 (0.7%)	25 (0.6%)
**SaO**_2_			
Median [IQR]	97.0 [94.0, 99.0]	97.0 [94.0, 99.0]	97.0 [94.0, 99.0]
Missing	34 (1.2%)	11 (1.1%)	45 (1.1%)
**BUN (mg/dL)**			
Median [IQR]	23.0 [16.0, 38.0]	29.0 [18.0, 49.0]	24.0 [16.0, 41.0]
Missing	124 (4.2%)	55 (5.3%)	179 (4.5%)
**Troponin-I**			
Median [IQR]	0.0200 [0.0100, 0.0500]	0.0500 [0.0200, 0.120]	0.0300 [0.0100, 0.0700]
Missing	558 (19.0%)	208 (20.2%)	766 (19.3%)
**On dialysis**			
No	2673 (90.9%)	928 (90.1%)	3601 (90.7%)
Yes	267 (9.1%)	102 (9.9%)	369 (9.3%)
**On supplemental O**_2_			
No	2876 (97.8%)	1006 (97.7%)	3882 (97.8%)
Yes	64 (2.2%)	24 (2.3%)	88 (2.2%)
**On ACE inhibitor**			
No	2391 (81.3%)	856 (83.1%)	3247 (81.8%)
Yes	549 (18.7%)	174 (16.9%)	723 (18.2%)
**Prolonged QRS duration**			
< = 120	1853 (63.0%)	643 (62.4%)	2496 (62.9%)
> 120	970 (33.0%)	369 (35.8%)	1339 (33.7%)
Missing	117 (4.0%)	18 (1.7%)	135 (3.4%)

*Note*: Values are counts (proportions) or median (lower and upper quartiles).

Abbreviations: BNP, B-type natriuretic peptide; BUN, blood urea nitrogen; DBP, diastolic blood pressure; SaO_2_ , arterial oxygen saturation.

**TABLE 2:** The model performance of full STRATIFY model and eligible submodels corresponding to observed missing patterns.

	C-index	Calibration-in- the-large	Calibration slope	NPV (the 5th percentile)	Brier score	Missing frequency
Full model	0.62 (0.59-0.67)	−0.04 (−0.21-0.12)	0.93 (0.56-1.30)	0.90 (0.79-0.97)	0.19 (0.17-0.20)	1434
Submodel missing (eligible)						
BMI	0.62 (0.59-0.66)	−0.04 (−0.21-0.12)	0.92 (0.55-1.30)	0.87 (0.77-0.94)	0.19 (0.17-0.20)	196
BNP	0.62 (0.58-0.66)	−0.02 (−0.18-0.14)	0.90 (0.52-1.29)	0.87 (0.77-0.96)	0.19 (0.18-0.20)	316
DBP	0.62 (0.58-0.66)	−0.04 (−0.20-0.12)	0.92 (0.55-1.29)	0.90 (0.79-0.97)	0.19 (0.17-0.20)	14
RR	0.62 (0.58-0.66)	−0.04 (−0.21-0.12)	0.90 (0.52-1.27)	0.87 (0.78-0.97)	0.19 (0.17-0.20)	5
SaO_2_	0.62 (0.59-0.66)	−0.04 (−0.20-0.12)	0.92 (0.55-1.30)	0.90 (0.80-0.97)	0.19 (0.17-0.20)	9
BUN	0.62 (0.58-0.67)	−0.04 (−0.20-0.13)	0.94 (0.55-1.34)	0.84 (0.74-0.93)	0.19 (0.17-0.20)	2
Troponin	0.61 (0.58-0.65)	−0.05 (−0.21-0.12)	0.84 (0.47-1.20)	0.86 (0.76-0.94)	0.19 (0.17-0.20)	206
QRS (> 120 ms)	0.63 (0.59-0.67)	−0.05 (−0.21-0.11)	0.96 (0.58-1.34)	0.90 (0.80-0.97)	0.19 (0.17-0.20)	28
BMI, BNP	0.61 (0.57-0.64)	−0.02 (−0.18-0.14)	0.88 (0.48-1.27)	0.86 (0.75-0.94)	0.19 (0.18-0.20)	36
BMI, SaO_2_	0.62 (0.58-0.66)	−0.04 (−0.20-0.12)	0.92 (0.54-1.29)	0.87 (0.78-0.95)	0.19 (0.17-0.20)	2
BMI, QRS (> 120 ms)	0.63 (0.59-0.66)	−0.05 (−0.21-0.11)	0.96 (0.57-1.34)	0.89 (0.78-0.95)	0.19 (0.17-0.20)	1
BNP, RR	0.61 (0.57-0.65)	−0.02 (−0.18-0.14)	0.87 (0.47-1.26)	0.89 (0.77-0.97)	0.19 (0.18-0.20)	1
BNP, SaO_2_	0.61 (0.58-0.65)	−0.02 (−0.18-0.14)	0.90 (0.51-1.29)	0.88 (0.77-0.97)	0.19 (0.18-0.20)	2
BNP, BUN	0.61 (0.57-0.66)	−0.01 (−0.17-0.15)	0.92 (0.49-1.34)	0.88 (0.78-0.95)	0.19 (0.18-0.20)	1
BNP, QRS (> 120 ms)	0.62 (0.58-0.66)	−0.03 (−0.19-0.14)	0.93 (0.54-1.33)	0.90 (0.80-0.98)	0.19 (0.17-0.20)	7
DBP, SaO_2_	0.62 (0.58-0.66)	−0.04 (−0.20-0.13)	0.91 (0.54-1.28)	0.92 (0.81-0.97)	0.19 (0.17-0.20)	3
DBP, Troponin	0.61 (0.57-0.65)	−0.04 (−0.20-0.12)	0.83 (0.47-1.20)	0.89 (0.80-0.96)	0.19 (0.17-0.20)	5
Sodium, BUN	0.61 (0.57-0.66)	−0.03 (−0.19-0.13)	0.91 (0.50-1.31)	0.82 (0.73-0.92)	0.19 (0.17-0.20)	13
SaO_2_, Troponin	0.61 (0.57-0.65)	−0.04 (−0.21-0.12)	0.83 (0.46-1.20)	0.86 (0.77-0.93)	0.19 (0.17-0.20)	2
Troponin, QRS (> 120 ms)	0.61 (0.58-0.65)	−0.05 (−0.21-0.11)	0.87 (0.50-1.24)	0.88 (0.78-0.95)	0.19 (0.17-0.20)	12
BMI, BNP, QRS (> 120 ms)	0.61 (0.57-0.65)	−0.02 (−0.18-0.14)	0.91 (0.51-1.31)	0.89 (0.78-0.96)	0.19 (0.18-0.20)	1
DBP, Troponin, QRS (> 120 ms)	0.61 (0.57-0.65)	−0.05 (−0.21-0.12)	0.86 (0.49-1.24)	0.92 (0.83-0.98)	0.19 (0.17-0.20)	1
SaO_2_, Troponin, QRS (> 120 ms)	0.61 (0.57-0.65)	−0.05 (−0.21-0.12)	0.86 (0.48-1.23)	0.88 (0.80-0.95)	0.19 (0.17-0.20)	1
Submodel missing (non-eligible)						
BMI, Troponin	0.60 (0.57-0.65)	−0.04 (−0.21-0.12)	0.83 (0.46-1.19)	0.80 (0.71-0.91)	0.19 (0.17-0.20)	12
BNP, DBP	0.61 (0.58-0.65)	−0.02 (−0.18-0.14)	0.89 (0.50-1.28)	0.84 (0.73-0.96)	0.19 (0.18-0.20)	3
BNP, Troponin	0.59 (0.55-0.63)	−0.02 (−0.18-0.14)	0.73 (0.34-1.13)	0.84 (0.74-0.92)	0.19 (0.18-0.21)	286
BMI, BNP, Troponin	0.58 (0.54-0.61)	−0.01 (−0.17-0.15)	0.66 (0.25-1.06)	0.81 (0.70-0.91)	0.19 (0.18-0.21)	24
BMI, Troponin, QRS (> 120 ms)	0.61 (0.57-0.65)	−0.05 (−0.21-0.11)	0.85 (0.48-1.23)	0.84 (0.76-0.93)	0.19 (0.17-0.20)	1
BNP, DBP, Troponin	0.59 (0.55-0.63)	−0.02 (−0.18-0.14)	0.73 (0.34-1.13)	0.81 (0.73-0.92)	0.19 (0.18-0.21)	2
BNP, Sodium, BUN	0.60 (0.56-0.64)	−0.00 (−0.16-0.16)	0.86 (0.42-1.30)	0.83 (0.73-0.92)	0.19 (0.18-0.20)	13
BNP, RR, Troponin	0.58 (0.54-0.62)	−0.02 (−0.18-0.14)	0.67 (0.27-1.08)	0.84 (0.74-0.95)	0.19 (0.18-0.21)	1
BNP, SaO_2_, Troponin	0.59 (0.55-0.63)	−0.02 (−0.18-0.15)	0.72 (0.32-1.12)	0.83 (0.73-0.92)	0.19 (0.18-0.21)	3
BNP, Troponin, QRS (> 120 ms)	0.59 (0.55-0.63)	−0.02 (−0.18-0.14)	0.76 (0.36-1.15)	0.87 (0.77-0.95)	0.19 (0.18-0.21)	58
DBP, Sodium, BUN	0.61 (0.57-0.65)	−0.02 (−0.18-0.14)	0.89 (0.47-1.31)	0.91 (0.83-0.97)	0.19 (0.17-0.20)	1
DBP, RR, SaO_2_	0.61 (0.57-0.65)	−0.04 (−0.20-0.13)	0.88 (0.50-1.27)	0.88 (0.79-0.96)	0.19 (0.17-0.20)	6
Sodium, BUN, Troponin	0.59 (0.55-0.64)	−0.03 (−0.19-0.13)	0.74 (0.34-1.14)	0.79 (0.70-0.90)	0.19 (0.17-0.20)	3

*Note*: The submodel selection was based on the criteria of averaged NPV of 1st to 10th percentiles.

Abbreviations: BNP, B-type natriuretic peptide; BUN, blood urea nitrogen; CI, confidence interval; cr, cubic root; DBP, diastolic blood pressure; RR, respiratory rate; SaO_2_, arterial oxygen saturation.

## Data Availability

The authors are not authorized to share the electronic health records used in Section 4.
