# Supplementary material for: Addressing the implementation challenge of risk prediction model due to missing risk factors: The submodel approximation approach
Source: Stat Med. Author manuscript; Available in PMC 2026 Feb 19. (PMC12917879; doi:10.1002/sim.10184)
Supplement: Appendix S1: Supplemental Material. [file NIHMS2138675-supplement-Appendix_S1__Supplemental_Material_.pdf]

Supporting Information for "Addressing the Implementation Challenge of Risk Prediction Model due to Missing Risk Factors: the Submodel Approximation Approach" by

Tianyi Sun, Allison B. McCoy, Alan B. Storrow, Dandan Liu

Vanderbilt University Medical Center, Nashville, TN, 37203

Web Appendix A: Additional simulation setting

Table S1: True value coefficients for preconditioning outcome and distributions of risk factors in dataset 1

Risk Factors (X)	β^{pre}	Distribution
Intercept	2.77	N(0,1)
X_1	0.14	N(0,1)
X_2	-0.12	N(0,1)
X_3	0.5	N(0,1)
X_4	0.3	N(0,1)
X_5	-0.5	N(0,1)
X_6	-0.3	N(0,1)
X_7	0.15	N(0,1)
X_8	-0.12	N(0,1)
X_9	-0.15	N(0,1)
X_{10}	0.6	Bernoulli(0.05)
X_{11}	0.2	Bernoulli(0.05)
X_{12}	-0.6	Bernoulli(0.3)
X_{13}	-0.2	Bernoulli(0.3)

Table S2: Scenarios of heterogeneity in risk factor distributions, for dataset 2

Scenario	Continuous (X_3, X_4, X_5, X_6)	Categorical ($X_{10}, X_{11}, X_{12}, X_{13}$)
0	-	-
1	Mean increases to 1	-
2	Mean reduces to -1	-
3	Variance increases 20%	-
4	Variance reduces 20%	-
5	-	Prevalence increases 20%
6	-	Prevalence reduces 20%

Web Appendix B: Additional simulation results

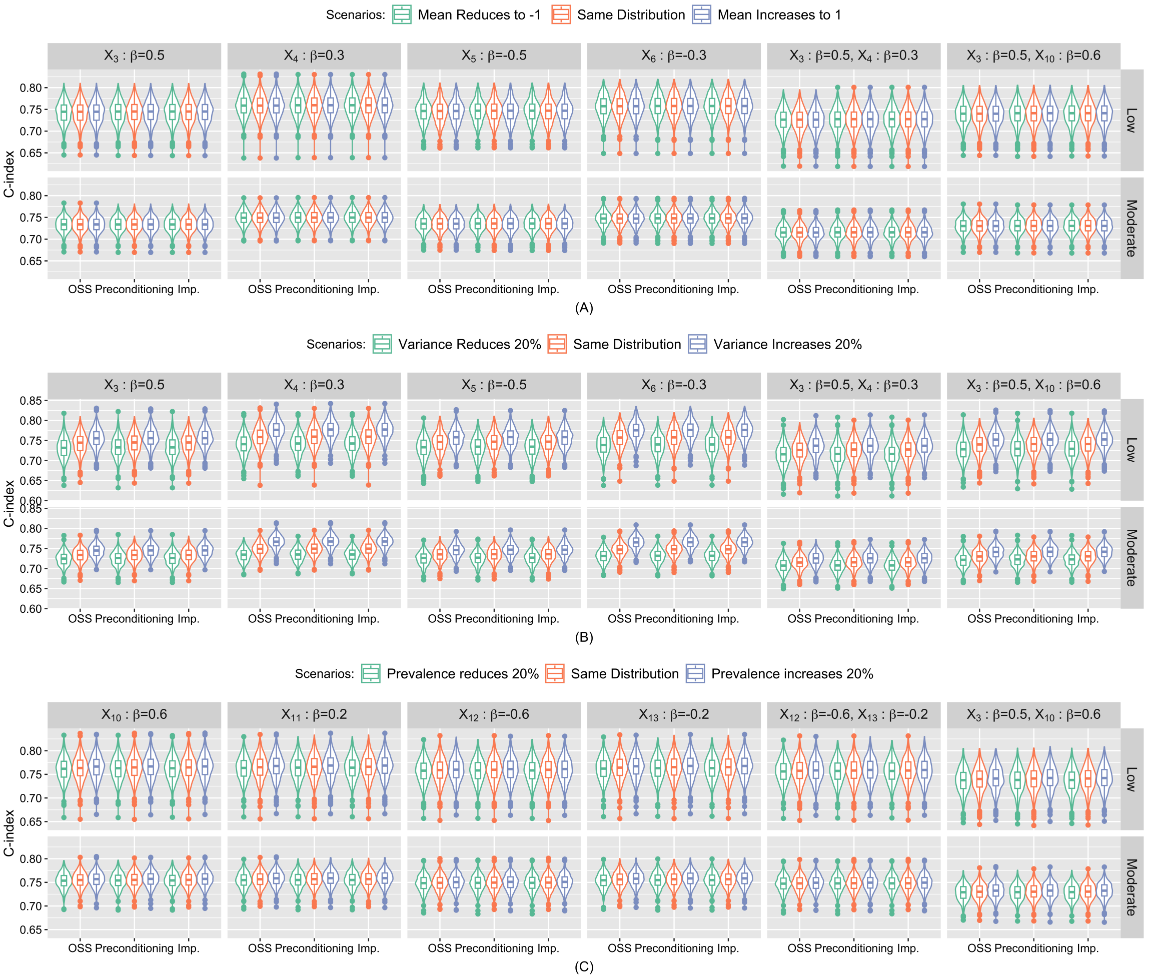

Figure S1: Simulation study results: Violin plots of C-index under three heterogeneity scenarios: (A) Heterogeneous mean, (B) heterogeneous variance and (C) heterogeneous prevalence from submodel- and imputation-based approaches corresponding to missing one or two continuous/binary risk factors. Each column represents missing risk factors with varying importance (standardized coefficient) in the risk calculation. X_3-X_6 represent continuous risk factors, while X_{10}-X_{13} represent binary risk factors, respectively. The first row represents scenarios with low event rate (10%). The second row represents scenarios with moderate event rate (30%).

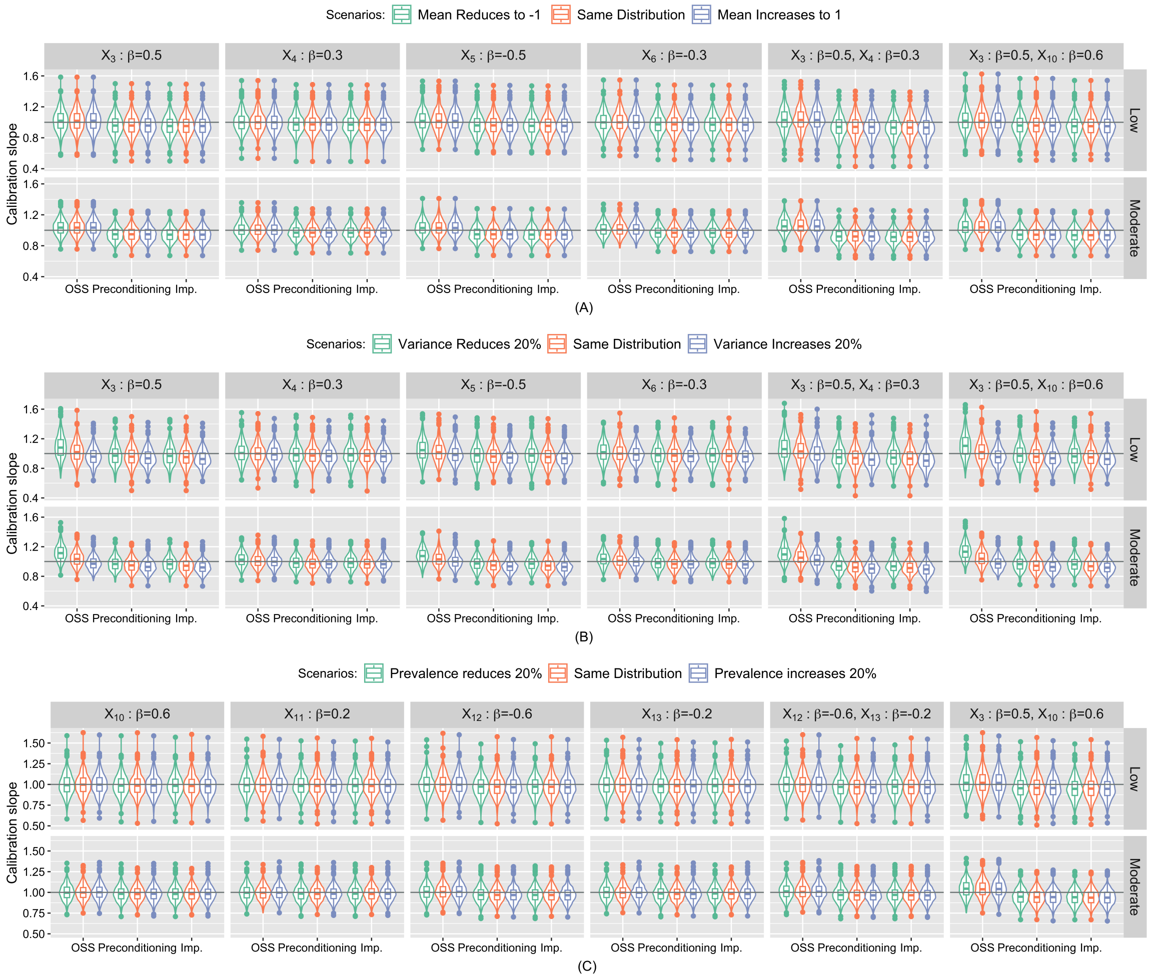

Figure S2: Simulation study results: Violin plots of calibration slope under three heterogeneity scenarios: (A) Heterogeneous mean, (B) heterogeneous variance and (C) heterogeneous prevalence from submodel- and imputation-based approaches corresponding to missing one or two continuous/binary risk factors. Each column represents missing risk factors with varying importance (standardized coefficient) in the risk calculation. X_3-X_6 represent continuous risk factors, while X_{10}-X_{13} represent binary risk factors, respectively. The first row represents scenarios with low event rate (10%). The second row represents scenarios with moderate event rate (30%). The grey horizontal line at one represents the ideal calibration slope.

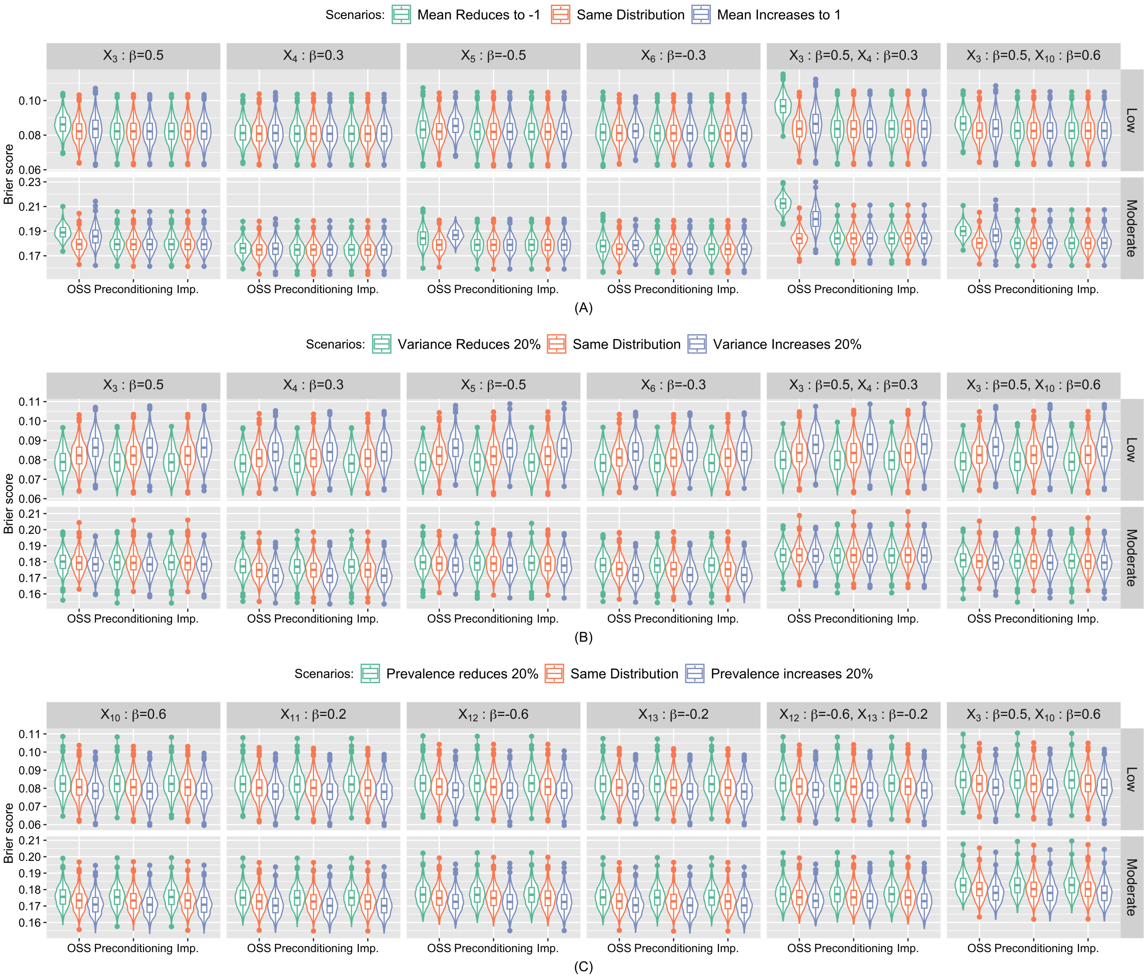

Figure S3: Simulation study results: Violin plots of Brier score under three heterogeneity scenarios: (A) Heterogeneous mean, (B) heterogeneous variance and (C) heterogeneous prevalence from submodel- and imputation-based approaches corresponding to missing one or two continuous/binary risk factors. Each column represents missing risk factors with varying importance (standardized coefficient) in the risk calculation. X_3-X_6 represent continuous risk factors, while X_{10}-X_{13} represent binary risk factors, respectively. The first row represents scenarios with low event rate (10%). The second row represents scenarios with moderate event rate (30%).

Web Appendix C: Additional application steps

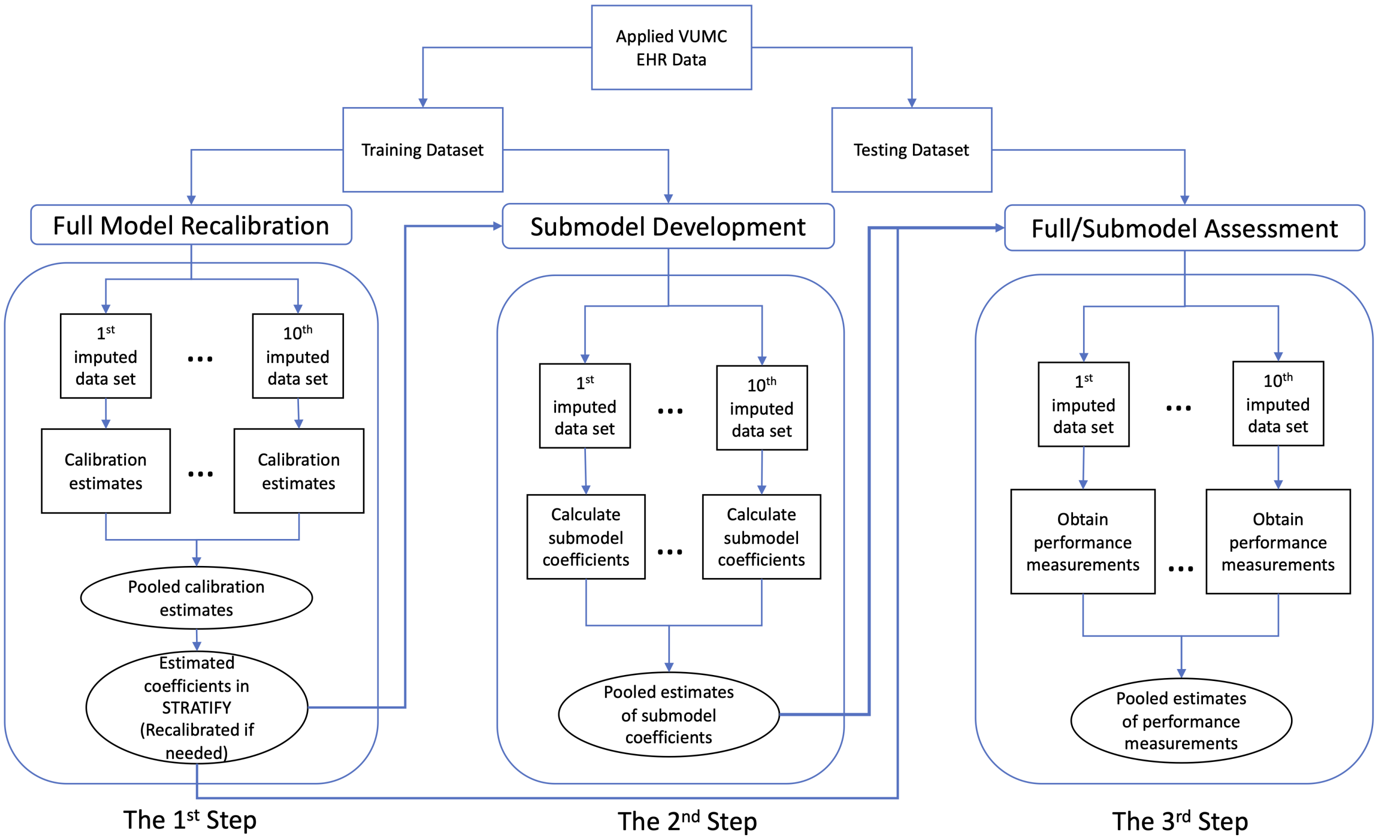

Figure S4: The flow of developing and assessing the proposed submodel based on multiple imputation. The step of full/submodel assessment was replicated 100 times using bootstrap samples

Web Appendix D: Additional application results

Table S3: The performance of full STRATIFY model and 129 preconditioning-based submodels

	C-index	Calibration-in-the-large	Calibration slope	NPV (the 5th Percentile)	Brier score	Missing Frequency
Full model	0.62(0.59-0.67)	-0.04(-0.21-0.12)	0.93(0.56-1.30)	0.90(0.79-0.97)	0.19(0.17-0.20)	1434
BMI	0.62(0.59-0.66)	-0.04(-0.21-0.12)	0.92(0.55-1.30)	0.87(0.77-0.94)	0.19(0.17-0.20)	196
BNP	0.62(0.58-0.66)	-0.02(-0.18-0.14)	0.90(0.52-1.29)	0.87(0.77-0.96)	0.19(0.18-0.20)	316
DBP	0.62(0.58-0.66)	-0.04(-0.20-0.12)	0.92(0.55-1.29)	0.90(0.79-0.97)	0.19(0.17-0.20)	14
Sodium	0.62(0.58-0.66)	-0.04(-0.20-0.12)	0.89(0.52-1.27)	0.88(0.77-0.96)	0.19(0.17-0.20)	0
RR	0.62(0.58-0.66)	-0.04(-0.21-0.12)	0.90(0.52-1.27)	0.87(0.78-0.97)	0.19(0.17-0.20)	5
SaO_2	0.62(0.59-0.66)	-0.04(-0.20-0.12)	0.92(0.55-1.30)	0.90(0.80-0.97)	0.19(0.17-0.20)	9
BUN	0.62(0.58-0.67)	-0.04(-0.20-0.13)	0.94(0.55-1.34)	0.84(0.74-0.93)	0.19(0.17-0.20)	2
Troponin	0.61(0.58-0.65)	-0.05(-0.21-0.12)	0.84(0.47-1.20)	0.86(0.76-0.94)	0.19(0.17-0.20)	206
QRS (>120 ms)	0.63(0.59-0.67)	-0.05(-0.21-0.11)	0.96(0.58-1.34)	0.90(0.80-0.97)	0.19(0.17-0.20)	28
BMI, BNP	0.61(0.57-0.64)	-0.02(-0.18-0.14)	0.88(0.48-1.27)	0.86(0.75-0.94)	0.19(0.18-0.20)	36
BMI, DBP	0.62(0.58-0.66)	-0.04(-0.20-0.12)	0.91(0.54-1.28)	0.88(0.81-0.97)	0.19(0.17-0.20)	0
BMI, Sodium	0.62(0.58-0.66)	-0.04(-0.20-0.13)	0.89(0.51-1.27)	0.90(0.79-0.96)	0.19(0.17-0.20)	0
BMI, RR	0.62(0.58-0.66)	-0.04(-0.21-0.12)	0.90(0.52-1.27)	0.84(0.74-0.93)	0.19(0.17-0.20)	0
BMI, SaO_2	0.62(0.58-0.66)	-0.04(-0.20-0.12)	0.92(0.54-1.29)	0.87(0.78-0.95)	0.19(0.17-0.20)	2
BMI, BUN	0.62(0.58-0.67)	-0.04(-0.20-0.13)	0.94(0.54-1.34)	0.83(0.74-0.91)	0.19(0.17-0.20)	0
BMI, Troponin	0.60(0.57-0.65)	-0.04(-0.21-0.12)	0.83(0.46-1.19)	0.80(0.71-0.91)	0.19(0.17-0.20)	12
BMI, QRS (>120 ms)	0.63(0.59-0.66)	-0.05(-0.21-0.11)	0.96(0.57-1.34)	0.89(0.78-0.95)	0.19(0.17-0.20)	1
BNP, DBP	0.61(0.58-0.65)	-0.02(-0.18-0.14)	0.89(0.50-1.28)	0.84(0.73-0.96)	0.19(0.18-0.20)	3
BNP, Sodium	0.61(0.58-0.65)	-0.02(-0.18-0.15)	0.85(0.47-1.24)	0.85(0.73-0.94)	0.19(0.17-0.20)	0
BNP, RR	0.61(0.57-0.65)	-0.02(-0.18-0.14)	0.87(0.47-1.26)	0.89(0.77-0.97)	0.19(0.18-0.20)	1
BNP, SaO_2	0.61(0.58-0.65)	-0.02(-0.18-0.14)	0.90(0.51-1.29)	0.88(0.77-0.97)	0.19(0.18-0.20)	2
BNP, BUN	0.61(0.57-0.66)	-0.01(-0.17-0.15)	0.92(0.49-1.34)	0.88(0.78-0.95)	0.19(0.18-0.20)	1
BNP, Troponin	0.59(0.55-0.63)	-0.02(-0.18-0.14)	0.73(0.34-1.13)	0.84(0.74-0.92)	0.19(0.18-0.21)	286
BNP, QRS (>120 ms)	0.62(0.58-0.66)	-0.03(-0.19-0.14)	0.93(0.54-1.33)	0.90(0.80-0.98)	0.19(0.17-0.20)	7
DBP, Sodium	0.62(0.58-0.65)	-0.03(-0.19-0.13)	0.88(0.50-1.25)	0.87(0.77-0.95)	0.19(0.17-0.20)	0
DBP, RR	0.62(0.58-0.66)	-0.04(-0.20-0.12)	0.89(0.51-1.27)	0.89(0.79-0.96)	0.19(0.17-0.20)	0
DBP, SaO_2	0.62(0.58-0.66)	-0.04(-0.20-0.13)	0.91(0.54-1.28)	0.92(0.81-0.97)	0.19(0.17-0.20)	3
DBP, BUN	0.61(0.58-0.67)	-0.03(-0.19-0.13)	0.94(0.53-1.34)	0.91(0.83-0.98)	0.19(0.17-0.20)	0
DBP, Troponin	0.61(0.57-0.65)	-0.04(-0.20-0.12)	0.83(0.47-1.20)	0.89(0.80-0.96)	0.19(0.17-0.20)	5
DBP, QRS (>120 ms)	0.63(0.59-0.67)	-0.04(-0.21-0.12)	0.95(0.57-1.33)	0.92(0.83-0.97)	0.19(0.17-0.20)	0
Sodium, RR	0.61(0.58-0.65)	-0.04(-0.20-0.12)	0.86(0.48-1.24)	0.86(0.76-0.95)	0.19(0.17-0.20)	0
Sodium, SaO_2	0.62(0.58-0.65)	-0.04(-0.20-0.13)	0.89(0.51-1.27)	0.89(0.80-0.97)	0.19(0.17-0.20)	0
Sodium, BUN	0.61(0.57-0.66)	-0.03(-0.19-0.13)	0.91(0.50-1.31)	0.82(0.73-0.92)	0.19(0.17-0.20)	13
Sodium, Troponin	0.60(0.57-0.64)	-0.04(-0.20-0.12)	0.80(0.43-1.17)	0.85(0.75-0.93)	0.19(0.17-0.20)	0
Sodium, QRS (>120 ms)	0.62(0.59-0.66)	-0.04(-0.21-0.12)	0.92(0.54-1.31)	0.88(0.78-0.97)	0.19(0.17-0.20)	0
RR, SaO_2	0.61(0.57-0.65)	-0.04(-0.20-0.12)	0.89(0.51-1.28)	0.87(0.78-0.95)	0.19(0.17-0.20)	0
RR, BUN	0.61(0.58-0.67)	-0.04(-0.20-0.12)	0.91(0.51-1.31)	0.88(0.77-0.94)	0.19(0.17-0.20)	0
RR, Troponin	0.60(0.56-0.64)	-0.05(-0.21-0.12)	0.80(0.43-1.17)	0.84(0.73-0.92)	0.19(0.17-0.20)	0
RR, QRS (>120 ms)	0.62(0.58-0.66)	-0.05(-0.21-0.11)	0.93(0.55-1.31)	0.90(0.79-0.97)	0.19(0.17-0.20)	0
SaO_2, BUN	0.62(0.58-0.66)	-0.03(-0.20-0.13)	0.94(0.55-1.34)	0.89(0.79-0.95)	0.19(0.17-0.20)	0
SaO_2, Troponin	0.61(0.57-0.65)	-0.04(-0.21-0.12)	0.83(0.46-1.20)	0.86(0.77-0.93)	0.19(0.17-0.20)	2
SaO_2, QRS (>120 ms)	0.63(0.59-0.67)	-0.05(-0.21-0.12)	0.96(0.58-1.34)	0.93(0.84-0.97)	0.19(0.17-0.20)	0
BUN, Troponin	0.59(0.56-0.65)	-0.04(-0.20-0.13)	0.80(0.41-1.18)	0.79(0.69-0.90)	0.19(0.17-0.20)	0
BUN, QRS (>120 ms)	0.63(0.59-0.67)	-0.04(-0.20-0.12)	0.99(0.58-1.40)	0.87(0.76-0.95)	0.19(0.17-0.20)	0

Table S3: The performance of full STRATIFY model and 129 preconditioning-based submodels (cont.)

Troponin, QRS (>120 ms)	0.61(0.58-0.65)	-0.05(-0.21-0.11)	0.87(0.50-1.24)	0.88(0.78-0.95)	0.19(0.17-0.20)	12
BMI, BNP, DBP	0.61(0.57-0.64)	-0.01(-0.18-0.15)	0.86(0.47-1.26)	0.84(0.72-0.93)	0.19(0.18-0.20)	0
BMI, BNP, Sodium	0.60(0.57-0.64)	-0.01(-0.17-0.15)	0.82(0.42-1.22)	0.86(0.76-0.95)	0.19(0.18-0.20)	0
BMI, BNP, RR	0.60(0.56-0.64)	-0.02(-0.18-0.14)	0.84(0.44-1.24)	0.82(0.73-0.94)	0.19(0.18-0.20)	0
BMI, BNP, SaO_2	0.61(0.57-0.64)	-0.01(-0.18-0.15)	0.87(0.47-1.27)	0.88(0.78-0.95)	0.19(0.18-0.20)	0
BMI, BNP, BUN	0.60(0.57-0.65)	-0.01(-0.17-0.16)	0.89(0.46-1.32)	0.86(0.75-0.94)	0.19(0.18-0.20)	0
BMI, BNP, Troponin	0.58(0.54-0.61)	-0.01(-0.17-0.15)	0.66(0.25-1.06)	0.81(0.70-0.91)	0.19(0.18-0.21)	24
BMI, BNP, QRS (>120 ms)	0.61(0.57-0.65)	-0.02(-0.18-0.14)	0.91(0.51-1.31)	0.89(0.78-0.96)	0.19(0.18-0.20)	1
BMI, DBP, Sodium	0.61(0.57-0.65)	-0.03(-0.19-0.13)	0.87(0.48-1.25)	0.86(0.75-0.95)	0.19(0.17-0.20)	0
BMI, DBP, RR	0.61(0.57-0.66)	-0.04(-0.20-0.12)	0.89(0.51-1.27)	0.86(0.77-0.95)	0.19(0.17-0.20)	0
BMI, DBP, SaO_2	0.62(0.58-0.65)	-0.04(-0.20-0.13)	0.90(0.53-1.28)	0.90(0.81-0.97)	0.19(0.17-0.20)	0
BMI, DBP, BUN	0.61(0.57-0.67)	-0.03(-0.19-0.13)	0.93(0.53-1.33)	0.90(0.81-0.98)	0.19(0.17-0.20)	0
BMI, DBP, Troponin	0.60(0.57-0.64)	-0.04(-0.20-0.12)	0.82(0.45-1.19)	0.88(0.80-0.97)	0.19(0.17-0.20)	0
BMI, DBP, QRS (>120 ms)	0.62(0.58-0.67)	-0.04(-0.21-0.12)	0.95(0.56-1.33)	0.89(0.81-0.97)	0.19(0.17-0.20)	0
BMI, Sodium, RR	0.61(0.57-0.65)	-0.04(-0.20-0.13)	0.86(0.47-1.24)	0.83(0.75-0.95)	0.19(0.17-0.20)	0
BMI, Sodium, SaO_2	0.61(0.57-0.65)	-0.03(-0.20-0.13)	0.88(0.50-1.26)	0.88(0.80-0.96)	0.19(0.17-0.20)	0
BMI, Sodium, BUN	0.61(0.57-0.66)	-0.03(-0.19-0.13)	0.90(0.49-1.31)	0.86(0.77-0.93)	0.19(0.17-0.20)	0
BMI, Sodium, Troponin	0.60(0.56-0.64)	-0.04(-0.20-0.12)	0.78(0.41-1.16)	0.82(0.71-0.93)	0.19(0.17-0.20)	0
BMI, Sodium, QRS (>120 ms)	0.62(0.58-0.66)	-0.04(-0.20-0.12)	0.92(0.53-1.31)	0.90(0.83-0.97)	0.19(0.17-0.20)	0
BMI, RR, SaO_2	0.61(0.57-0.65)	-0.04(-0.20-0.12)	0.89(0.50-1.27)	0.84(0.77-0.94)	0.19(0.17-0.20)	0
BMI, RR, BUN	0.61(0.57-0.67)	-0.04(-0.20-0.13)	0.91(0.51-1.31)	0.87(0.76-0.94)	0.19(0.17-0.20)	0
BMI, RR, Troponin	0.60(0.56-0.64)	-0.04(-0.21-0.12)	0.79(0.41-1.16)	0.80(0.67-0.93)	0.19(0.17-0.20)	0
BMI, RR, QRS (>120 ms)	0.62(0.58-0.66)	-0.05(-0.21-0.12)	0.93(0.54-1.31)	0.86(0.76-0.95)	0.19(0.17-0.20)	0
BMI, SaO_2, BUN	0.61(0.57-0.66)	-0.03(-0.20-0.13)	0.94(0.54-1.34)	0.84(0.74-0.92)	0.19(0.17-0.20)	0
BMI, SaO_2, Troponin	0.60(0.57-0.64)	-0.04(-0.21-0.12)	0.82(0.45-1.18)	0.83(0.75-0.91)	0.19(0.17-0.20)	0
BMI, SaO_2, QRS (>120 ms)	0.63(0.59-0.66)	-0.05(-0.21-0.12)	0.95(0.57-1.33)	0.89(0.80-0.95)	0.19(0.17-0.20)	0
BMI, BUN, Troponin	0.59(0.55-0.64)	-0.04(-0.20-0.13)	0.79(0.39-1.18)	0.76(0.67-0.88)	0.19(0.17-0.20)	0
BMI, BUN, QRS (>120 ms)	0.62(0.59-0.67)	-0.04(-0.20-0.12)	0.99(0.58-1.40)	0.88(0.76-0.95)	0.19(0.17-0.20)	0
BMI, Troponin, QRS (>120 ms)	0.61(0.57-0.65)	-0.05(-0.21-0.11)	0.85(0.48-1.23)	0.84(0.76-0.93)	0.19(0.17-0.20)	1
BNP, DBP, Sodium	0.61(0.57-0.65)	-0.01(-0.17-0.15)	0.84(0.44-1.23)	0.83(0.73-0.94)	0.19(0.18-0.20)	0
BNP, DBP, RR	0.61(0.57-0.65)	-0.02(-0.18-0.14)	0.86(0.46-1.25)	0.89(0.79-0.96)	0.19(0.18-0.20)	0
BNP, DBP, SaO_2	0.61(0.57-0.65)	-0.02(-0.18-0.15)	0.89(0.49-1.28)	0.90(0.79-0.97)	0.19(0.18-0.20)	0
BNP, DBP, BUN	0.60(0.57-0.66)	-0.00(-0.16-0.16)	0.90(0.47-1.34)	0.85(0.76-0.95)	0.19(0.18-0.20)	0
BNP, DBP, Troponin	0.59(0.55-0.63)	-0.02(-0.18-0.14)	0.73(0.34-1.13)	0.81(0.73-0.92)	0.19(0.18-0.21)	2
BNP, DBP, QRS (>120 ms)	0.62(0.58-0.66)	-0.02(-0.19-0.14)	0.92(0.53-1.32)	0.90(0.79-0.97)	0.19(0.17-0.20)	0
BNP, Sodium, RR	0.60(0.56-0.64)	-0.02(-0.18-0.15)	0.81(0.42-1.21)	0.85(0.74-0.94)	0.19(0.18-0.20)	0

Table S3: The performance of full STRATIFY model and 129 preconditioning-based submodels (cont.)

BNP, Sodium, SaO_2	0.61(0.57-0.65)	-0.01(-0.17-0.15)	0.85(0.46-1.25)	0.85(0.74-0.95)	0.19(0.18-0.20)	0
BNP, Sodium, BUN	0.60(0.56-0.64)	-0.00(-0.16-0.16)	0.86(0.42-1.30)	0.83(0.73-0.92)	0.19(0.18-0.20)	13
BNP, Sodium, Troponin	0.58(0.54-0.62)	-0.01(-0.17-0.15)	0.68(0.28-1.07)	0.80(0.67-0.91)	0.19(0.18-0.21)	0
BNP, Sodium, QRS (>120 ms)	0.61(0.58-0.65)	-0.02(-0.18-0.14)	0.88(0.49-1.28)	0.88(0.77-0.96)	0.19(0.17-0.20)	0
BNP, RR, SaO_2	0.60(0.56-0.64)	-0.02(-0.18-0.14)	0.86(0.46-1.27)	0.88(0.76-0.97)	0.19(0.18-0.20)	0
BNP, RR, BUN	0.60(0.56-0.65)	-0.01(-0.17-0.15)	0.87(0.44-1.30)	0.84(0.76-0.93)	0.19(0.18-0.20)	0
BNP, RR, Troponin	0.58(0.54-0.62)	-0.02(-0.18-0.14)	0.67(0.27-1.08)	0.84(0.74-0.95)	0.19(0.18-0.21)	1
BNP, RR, QRS (>120 ms)	0.61(0.57-0.65)	-0.03(-0.19-0.14)	0.89(0.50-1.29)	0.89(0.78-0.97)	0.19(0.18-0.20)	0
BNP, SaO_2, BUN	0.60(0.57-0.65)	-0.01(-0.17-0.15)	0.92(0.49-1.35)	0.87(0.79-0.95)	0.19(0.18-0.20)	0
BNP, SaO_2, Troponin	0.59(0.55-0.63)	-0.02(-0.18-0.15)	0.72(0.32-1.12)	0.83(0.73-0.92)	0.19(0.18-0.21)	3
BNP, SaO_2, QRS (>120 ms)	0.62(0.58-0.65)	-0.02(-0.18-0.14)	0.93(0.53-1.33)	0.88(0.79-0.97)	0.19(0.18-0.20)	0
BNP, BUN, Troponin	0.56(0.52-0.61)	-0.00(-0.16-0.16)	0.58(0.13-1.03)	0.75(0.61-0.87)	0.19(0.18-0.21)	0
BNP, BUN, QRS (>120 ms)	0.61(0.57-0.66)	-0.01(-0.18-0.15)	0.97(0.53-1.41)	0.86(0.77-0.95)	0.19(0.18-0.20)	0
BNP, Troponin, QRS (>120 ms)	0.59(0.55-0.63)	-0.02(-0.18-0.14)	0.76(0.36-1.15)	0.87(0.77-0.95)	0.19(0.18-0.21)	58
DBP, Sodium, RR	0.61(0.57-0.65)	-0.03(-0.20-0.13)	0.85(0.46-1.23)	0.85(0.74-0.93)	0.19(0.17-0.20)	0
DBP, Sodium, SaO_2	0.61(0.58-0.65)	-0.03(-0.19-0.13)	0.87(0.49-1.25)	0.89(0.79-0.95)	0.19(0.17-0.20)	0
DBP, Sodium, BUN	0.61(0.57-0.65)	-0.02(-0.18-0.14)	0.89(0.47-1.31)	0.91(0.83-0.97)	0.19(0.17-0.20)	1
DBP, Sodium, Troponin	0.60(0.56-0.64)	-0.03(-0.20-0.13)	0.79(0.42-1.16)	0.88(0.79-0.95)	0.19(0.17-0.20)	0
DBP, Sodium, QRS (>120 ms)	0.62(0.58-0.65)	-0.04(-0.20-0.13)	0.91(0.52-1.30)	0.90(0.81-0.96)	0.19(0.17-0.20)	0
DBP, RR, SaO_2	0.61(0.57-0.65)	-0.04(-0.20-0.13)	0.88(0.50-1.27)	0.88(0.79-0.96)	0.19(0.17-0.20)	6
DBP, RR, BUN	0.61(0.57-0.66)	-0.03(-0.19-0.13)	0.90(0.50-1.31)	0.89(0.81-0.97)	0.19(0.17-0.20)	0
DBP, RR, Troponin	0.60(0.56-0.64)	-0.04(-0.20-0.12)	0.79(0.42-1.17)	0.87(0.77-0.95)	0.19(0.17-0.20)	0
DBP, RR, QRS (>120 ms)	0.62(0.58-0.66)	-0.04(-0.21-0.12)	0.92(0.54-1.31)	0.91(0.81-0.97)	0.19(0.17-0.20)	0
DBP, SaO_2, BUN	0.61(0.57-0.66)	-0.03(-0.19-0.14)	0.93(0.52-1.34)	0.91(0.83-0.98)	0.19(0.17-0.20)	0
DBP, SaO_2, Troponin	0.60(0.57-0.64)	-0.04(-0.20-0.12)	0.82(0.45-1.19)	0.91(0.82-0.97)	0.19(0.17-0.20)	0
DBP, SaO_2, QRS (>120 ms)	0.63(0.58-0.66)	-0.04(-0.20-0.12)	0.95(0.56-1.33)	0.92(0.85-0.98)	0.19(0.17-0.20)	0
DBP, BUN, Troponin	0.59(0.55-0.64)	-0.03(-0.19-0.13)	0.78(0.39-1.18)	0.88(0.78-0.95)	0.19(0.18-0.20)	0
DBP, BUN, QRS (>120 ms)	0.62(0.58-0.68)	-0.03(-0.20-0.13)	0.99(0.57-1.42)	0.91(0.83-0.98)	0.19(0.17-0.20)	0
DBP, Troponin, QRS (>120 ms)	0.61(0.57-0.65)	-0.05(-0.21-0.12)	0.86(0.49-1.24)	0.92(0.83-0.98)	0.19(0.17-0.20)	1
Sodium, RR, SaO_2	0.61(0.57-0.65)	-0.03(-0.20-0.13)	0.86(0.47-1.25)	0.86(0.77-0.95)	0.19(0.17-0.20)	0
Sodium, RR, BUN	0.61(0.57-0.66)	-0.03(-0.19-0.13)	0.87(0.46-1.28)	0.83(0.71-0.93)	0.19(0.17-0.20)	0
Sodium, RR, Troponin	0.60(0.56-0.64)	-0.04(-0.20-0.12)	0.76(0.38-1.13)	0.86(0.75-0.94)	0.19(0.17-0.21)	0
Sodium, RR, QRS (>120 ms)	0.62(0.58-0.65)	-0.04(-0.21-0.12)	0.89(0.50-1.28)	0.87(0.77-0.96)	0.19(0.17-0.20)	0
Sodium, SaO_2, BUN	0.61(0.57-0.66)	-0.03(-0.19-0.14)	0.91(0.50-1.32)	0.87(0.76-0.94)	0.19(0.17-0.20)	0

Table S3: The performance of full STRATIFY model and 129 preconditioning-based submodels (cont.)

Sodium, SaO_2, Troponin	0.60(0.56-0.64)	-0.04(-0.20-0.13)	0.79(0.42-1.17)	0.85(0.77-0.93)	0.19(0.17-0.20)	0
Sodium, SaO_2, QRS (>120 ms)	0.62(0.58-0.66)	-0.04(-0.20-0.12)	0.92(0.54-1.31)	0.91(0.82-0.97)	0.19(0.17-0.20)	0
Sodium, BUN, Troponin	0.59(0.55-0.64)	-0.03(-0.19-0.13)	0.74(0.34-1.14)	0.79(0.70-0.90)	0.19(0.17-0.20)	3
Sodium, BUN, QRS (>120 ms)	0.62(0.58-0.67)	-0.03(-0.20-0.13)	0.96(0.53-1.38)	0.85(0.75-0.92)	0.19(0.17-0.20)	0
Sodium, Troponin, QRS (>120 ms)	0.61(0.57-0.65)	-0.04(-0.21-0.12)	0.83(0.45-1.21)	0.86(0.76-0.94)	0.19(0.17-0.20)	0
RR, SaO_2, BUN	0.61(0.57-0.66)	-0.03(-0.20-0.13)	0.91(0.50-1.31)	0.83(0.75-0.94)	0.19(0.17-0.20)	0
RR, SaO_2, Troponin	0.59(0.55-0.63)	-0.04(-0.20-0.12)	0.78(0.40-1.16)	0.85(0.77-0.95)	0.19(0.17-0.20)	0
RR, SaO_2, QRS (>120 ms)	0.62(0.58-0.65)	-0.04(-0.21-0.12)	0.92(0.53-1.32)	0.89(0.80-0.97)	0.19(0.17-0.20)	0
RR, BUN, Troponin	0.59(0.54-0.65)	-0.04(-0.20-0.13)	0.74(0.35-1.14)	0.79(0.66-0.90)	0.19(0.18-0.20)	0
RR, BUN, QRS (>120 ms)	0.62(0.58-0.67)	-0.04(-0.20-0.12)	0.95(0.54-1.37)	0.87(0.78-0.95)	0.19(0.17-0.20)	0
RR, Troponin, QRS (>120 ms)	0.60(0.57-0.64)	-0.05(-0.21-0.11)	0.82(0.45-1.20)	0.86(0.76-0.95)	0.19(0.17-0.20)	0
SaO_2, BUN, Troponin	0.59(0.56-0.64)	-0.03(-0.20-0.13)	0.79(0.39-1.18)	0.79(0.71-0.90)	0.19(0.17-0.20)	0
SaO_2, BUN, QRS (>120 ms)	0.62(0.59-0.67)	-0.04(-0.20-0.12)	0.99(0.58-1.41)	0.90(0.80-0.97)	0.19(0.17-0.20)	0
SaO_2, Troponin, QRS (>120 ms)	0.61(0.57-0.65)	-0.05(-0.21-0.12)	0.86(0.48-1.23)	0.88(0.80-0.95)	0.19(0.17-0.20)	1
BUN, Troponin, QRS (>120 ms)	0.60(0.57-0.65)	-0.04(-0.20-0.12)	0.84(0.43-1.24)	0.79(0.70-0.91)	0.19(0.17-0.20)	0

CI = confidence interval, SaO_2 = arterial oxygen saturation, cr = cubic root, DBP = diastolic blood pressure, RR= Respiratory Rate, BNP = B-type natriuretic peptide, BUN = blood urea nitrogen
